# Evolution of a structured cell population endowed with plasticity of traits under constraints on and between the traits

**DOI:** 10.1007/s00285-022-01820-5

**Published:** 2022-11-04

**Authors:** Frank Ernesto Alvarez, José Antonio Carrillo, Jean Clairambault

**Affiliations:** 1 CEREMADE (CNRS UMR no. 7534), PSL University, Université Paris - Dauphine, Place du Maréchal De Lattre De Tassigny, 75775 Paris CEDEX 16, France; 2 Mathematical Institute, University of Oxford, Oxford, OX2 6GG UK; 3 Laboratoire Jacques-Louis Lions and Inria Mamba team, Sorbonne Université, 4, place Jussieu, F75252 Paris CEDEX 05, France

**Keywords:** Structured population, Plasticity, Dimorphism, Finite volume method, 35K57, 65N08, 92B99

## Abstract

Confronted with the biological problem of managing plasticity in cell populations, which is in particular responsible for transient and reversible drug resistance in cancer, we propose a rationale consisting of an integro-differential and a reaction-advection-diffusion equation, the properties of which are studied theoretically and numerically. By using a constructive finite volume method, we show the existence and uniqueness of a weak solution and illustrate by numerical approximations and their simulations the capacity of the model to exhibit divergence of traits. This feature may be theoretically interpreted as describing a physiological step towards multicellularity in animal evolution and, closer to present-day clinical challenges in oncology, as a possible representation of bet hedging in cancer cell populations.

## Introduction: biological background

One of the main theories explaining the origins of cancer states that the hallmark capabilities of cancer are based on latent functions already existing in the genome of normal human cells, and that cancer represents a reversion to a less differentiated and less cooperative cellular behavior. This theory is usually called the atavistic model of cancer (Davies and Lineweaver 2011). It is opposed to the more commonly admitted somatic mutation theory (Lineweaver and Davies 2020), that states that cancer originated in a single cell, following a catastrophic sequence of stochastic tumorigenic mutations, and from which Darwinian selection produced by divisions from this unicellular basis a more or less organised society of cheating cells, more and more escaping controls by the host organism, i.e., a cancer. In the atavistic theory, accompanied or induced by blockade of differentiation or reverse differentiation of normally maturing cells, societies of cells in a multicellular organism (cancer is always a disease of multicellular organisms) somehow, in some location of the organism, escape the fine control under which they are normally placed and revert to a previous, coarse and disorganised state of multicellularity (Davies and Lineweaver 2011). This may be understood as a process of “deDarwinisation”, through which cancer cells gain a state of *plasticity* (Clairambault 2020; Shen and Clairambault 2020; Hanahan 2022; Yuan et al. 2019) representative of a former state in the evolution of multicellularity.

The atavistic theory has thus deep connections with the theory of evolution to multicellularity. The passage from unicellular organisms to multicellular ones led to the regulation of capabilities, resulting in controlled proliferation and differentiation of cells leading to specialisation and cooperation between specialised cells. The role of environment-driven cellular stress in this process of specialisation has recently been stressed by various authors (Nedelcu and Michod 2020; Wagner et al. 2019). The new genes responsible for these regulations became tumour suppressors. The atavistic theory states that if these new suppressors become damaged for some reason, then latent genes, associated with functions from unicellular organisms, will reappear and dominate the scenery, thus resulting in the unconstrained proliferation and the lack of cooperation with the other cells of the host organism, as actually found in tumours.

Understanding how evolution led to the emergence of multicellularity then becomes a problem closely related to that of understanding cancer. Firstly because unravelling in detail what is lost (cohesion) and what is gained (plasticity) in this reverse evolutionary process may help understand the nature of cancer from a functional point of view. Secondly because it is reasonable to assume that Darwinian selection in tumours starts from a primitive state of multicellularity in which cells are very plastic with respect to their phenotypes, which sends us back to states in evolution close to the emergence of multicellularity. The division of work through cell differentiation achieved by coherent multicellularity (i.e., designing a stable, cohesive multicellular organism) (Müller 2001) was of vital importance in order to evolve into more complex and more functionally efficient organisms. In a first stage, this differentiation was very likely reversible, due to the high plasticity that cells were endowed with in a primitive state of multicellularity. Under these conditions, one can reasonably assume that these primitive organisms adopted bet hedging strategies, i.e., common risk-diversifying strategies in unpredictably changing and often aggressive environments, in order to maximise their phenotypic fitness (Philippi and Seger 1989; Seger 1987).

In more detail, bet hedging in cell populations may be defined as a diversification of phenotypes in a cohesive (or at least bound by a common fate community, usually of genetic nature) cell population to optimise its fitness, in particular *a minima* to ensure its survival in a life-threatening environment, in other words to design a fail-safe strategy for the preservation of a propagating element; one cell may be enough to achieve this goal. Enhancing the ability to (quickly) diversify phenotypes by changing differentiation paths (by reversal, i.e., vertical de-differentiation, or by horizontal trandsdifferentiation in a differentiation tree) may in particular be achieved at the chromatin level by means of epigenetic enzymes, molecular instances of cell plasticity at work (Shen and Clairambault 2020).

Among such commonly described strategies of living organisms (unicellular or multicellular) meant to ensure survival in changing environments have been classically described fright, fight and flight. Fright (or freeze) is not likely to induce phenotype evolution. Fight (establishing barriers, secreting poisons, gathering in colonies) and flight (motility to escape unbeatable predators) can. Differentiation between somatic and germinal cells is also a major step in evolution. Bet hedging strategies were not only present at elementary stages of evolution. They are a common adaptive tool that can still be found in nature at different levels of complexity, from prokaryotic organisms to vertebrate ones. In between, tumour cells, thanks to their high plasticity, in the presence of an aggressive environment provided by immune response of the host body or of any anti-cancer treatment, may adopt bet hedging as a strategy to guarantee a prolonged survival of their colony. The wide presence of bet hedging in nature as an evolutionary mechanism, and its many links to the development of cancer is what motivates us in the present attempt towards a mathematical model representing some of the factors that influence this phenomenon (natural selection, epimutations and environmental stress).

In our modelling choices, we have privileged, for the sake of biological interpretation, as in Chisholm et al. (2015), two phenotypes that are often identified as such in theoretical ecology: viability (potential to resist a deadly insult: the elephant strategy) and fecundity (potential to proliferate, or in a way, surviving by numbers, even under hard environmental conditions: the rat strategy). They may influence cell behaviour in opposed directions, as two different choices, incompatible for the same cell, in the same way as fecundity and motility are notably incompatible (cells that divide do not move, and vice versa). To take into account the faculty of rapidly changing phenotypes in case of a life-threatening insult, a capacity reported about many cancer cells (e.g., epithelial to mesenchymal transition or drug-induced drug persistence) (Shen and Clairambault 2020), here we have added plasticity as a complementary structuring phenotype (or trait) of cells. Among questions at stake we will in particular deal with is the optimisation of fitness strategy by concentration of phenotypes, either in two or more ranges surrounding fixed points (multimodality of traits, i.e., bet hedging) or around a central optimal point (unimodality of trait, i.e. no bet hedging when it is not favourable).

## The model

We consider a population (not necessarily of tumour cells) in which each individual has three defining traits: viability associated with the variable $x\in [0,1]$ which reflects the potential to resist deadly insults, fecundity associated with the variable $y\in [0,1]$ representing the potential to proliferate and plasticity associated with the variable $\theta \in [0,1]$ which represents the potential to continue to differentiate (or de-differentiate, or transdifferentiate, as long as it has not been fixed at its lower bound $\theta$=0) within a differentiation tree. We assume furthermore that for a certain regular function $C:{\mathbb {R}}^2\rightarrow {\mathbb {R}}$ and a positive constant *K*, $(x,y)\in \Omega :=\{C(x,y)\leqslant K\}$, so $z=(x,y,\theta )$ ranges over the set $D:=\{\Omega \times [0,1] \}$. We then consider the evolution problem (1), (2), (3) on the density of population $n=n(t,z)\geqslant 0$.

1 $$\begin{aligned} \partial _t n+\nabla \cdot \Big (Vn-A(\theta )\nabla n\Big )&=(r(z)-d(z)\rho (t))n, \end{aligned}$$

2 $$\begin{aligned} \Big (Vn-A(\theta )\nabla n\Big )\cdot {\mathbf {n}}&=0, \text{ for } \text{ all } z\in \partial D,\end{aligned}$$

3 $$\begin{aligned} n(0,z)&=n_0(z), \text{ for } \text{ all } z\in D. \end{aligned}$$

In the above equation, chosen for the sake of simplicity as diagonal, the matrix

$$\begin{aligned} A(\theta )=\begin{pmatrix} a_{11}(\theta )&{}0&{}0\\ 0&{}a_{22}(\theta )&{}0\\ 0&{}0&{}a_{33} \end{pmatrix} \end{aligned}$$

gives the speed at which non-genetic epimutations occur, otherwise said it is a minimally simple representation of how the internal plasticity trait $\theta$ affects the non-genetic instability of traits *x* and *y*, by tuning the diffusion term; the function

$$\begin{aligned} V(t,z)=(V_1(t,z),V_2(t,z),V_3(t,z)) \end{aligned}$$

represents the sensitivity, meant here as the force of external evolutionary pressure, of the population to abrupt changes in the environment;

$$\begin{aligned} \rho (t)=\int \limits _{D}n(t,z)dz \end{aligned}$$

stands for the total amount of individuals in the population at time *t*.

From a biological point of view, the matrix $A(\theta )$ represents random epimutations leading to non genetic changes, increasing with plasticity $\theta$, in the traits viability *x* and fecundity *y*, while *V* stands for an external factor (abrupt biophysical changes in the ecosystem, exposure to life-threatening drugs in the case of cancer cell populations) inducing cellular stress in the population. Throughout the paper, we refer to $\nabla \cdot (Vn)$ as advection term, or drift term, indistinctly.

We chose the term $d(z)\rho (t)$ here as the simplest way to represent a death term in the evolution of the population. This multiplicative representation is classic, but most of the results we show in what follows can be extended to more general reaction terms.

Throughout our work we assume

1. For some $p>2$, the initial population density $n_0(z)$ belongs to $L^p(D)$.
2. The intrinsic growth rate in absence of competition *r*(*z*) and the death rate *d*(*z*) due to competition for individuals with trait *z* are positive bounded functions that satisfy $0<r^-\leqslant r(z)\leqslant r^+$ and $0<d^-\leqslant d(z)\leqslant d^+$.
3. The diffusion parameters $a_{11}(\theta )$, $a_{22}(\theta )$ and $a_{33}$ are strictly positive, with $a_{11}(\theta )$ and $a_{22}(\theta )$ being non decreasing with respect to $\theta$. Hence, the matrix $A(\theta )$ is elliptic for all values of $\theta$.
4. The function *V*(*t*, *z*) is continuously differentiable for all values of $t>0$ and $z\in D$.

Under these hypotheses we are able to prove the existence and uniqueness of a solution for the problem (1–3) using the finite volume method in order to obtain a convergent semi-discrete scheme.

The problem (1–3) underlying hypotheses *(H1)* to *(H4)* sets a structured population model of evolution that, as mentioned before, takes into account some of the factors that might lead to the occurrence of “bet hedging”. Amongst the first works in this topic, we can find (Cohen 1966), where is studied the fraction of seeds that germinate and the fraction that remains dormant, in order to maximise the long term expectation of growth. In the same work, the similarities of this phenomenon with economic decision making under risk (so-called “fail-safe strategies”) are noted. Other early works on the subject are Philippi and Seger (1989) and Seger (1987). The reaction term in (1) is a simple way of modelling the selection principle. This term and more general ones are used in Perthame (2006) in order to study some basic properties of structured populations undergoing this type of behaviour. The same reaction term is also used in Pouchol and Trélat (2018), where are analysed the global asymptotic stability properties for integro-differential systems of *N* species structured by different sets of traits. A similar competition term is used in Desvillettes et al. (2008) to provide results about the long time behaviour of such reaction models. The diffusion term here models non-genetic instabilities (also known as epimutations), which constitute the drift of phenotype without alteration of the genotype. In Perthame (2006) an integral operator in order to model mutations arising during reproduction is used, and something similar could be done for the epimutations. The second order operator used in (1) can be obtained then after re-scaling the time variable. The effect of this phenomenon in cancer development is discussed in Huang (2013) from a biological point of view. Epimutations can also occur because of external stress, and this is represented in (1) by means of the advection terms. A biological example for a population changing phenotype due to external stress can be found in Matuła et al. (2019), where the effect of physical stress on the shape and the cell wall thickness of E.coli bacterias is discussed. Two different models are used in Chisholm et al. (2015) to conclude that the three mechanisms described above might reversibly push an actively-proliferating, and drug-sensitive, cell population to transition into a weakly-proliferative and drug-tolerant state, which will eventually facilitate the emergence of more potent, proliferating and drug-tolerant cells. One of such models is an integro-differential model very similar to (1–3), but without including the effect of plasticity on the evolution of the population and without assuming the existence of a constraint between the traits *x* and *y*.

The main results of this paper involve the variational formulation of (1–3), which we now introduce. Denote $H=L^2(D)$, with $(\cdot ,\cdot )_H$ the usual scalar product in that space, and ${\mathcal {V}}=H^1(D)$ with $\langle \cdot ,\cdot \rangle =\langle \cdot ,\cdot \rangle _{{\mathcal {V}}'\times {\mathcal {V}}}$ being the duality product in ${\mathcal {V}}$.

For any given $n_0\in H$, $T>0$, we say that

$$\begin{aligned} n:=n(t)\in X_T:=C([0,T],H)\cap L^2((0,T),{\mathcal {V}})\cap H^1([0,T],{\mathcal {V}}'), \end{aligned}$$

is a variational solution of the problem (1–3) if it is a solution in the following weak sense

4 $$\begin{aligned} (n(t),\varphi (t))_H=&(n_0,\varphi (0))_H+\int _0^t \Big (\langle Q[n](s),\varphi (s)\rangle +\langle {\partial _s\varphi (s)},n(s)\rangle \Big )ds, \end{aligned}$$

where

$$\begin{aligned} \langle Q[n],\varphi \rangle =\int \limits _{D}\Big (-A\nabla n\nabla \varphi +Vn\nabla \varphi +(r(z)-\rho d(z))n\varphi \Big ) dz, \end{aligned}$$

for any $\varphi \in X_T$. We say that *n* is a global solution if it is a solution on [0, *T*] for any $T>0$.

### Theorem 1

For all non-negative $n_0\in L^p(D)$, $p>2$, there exists a unique global non-negative weak solution for problem (1–3) in the sense of (4).

We focus on giving a proof for this theorem using a discretised version of problem (1–3) after applying the Finite Volume Method to it. For this purpose, we define a set $D_h\supset D$, that can be covered by the union of *N* disjoint cubic cells, denoted as $D_j$, of side length *h*. After integrating the Eq. (1) over each of the cells $D_j$ we derive the system of first-order differential equations

5 $$\begin{aligned} \frac{d}{dt}\nu _j(t)=&M_{j}(t,\nu (t))\nu _j(t)+\sum \limits _{l\in N_j}B_{jl}(t)\nu _l(t), \end{aligned}$$

6 $$\begin{aligned} \nu _j(0)=&\frac{1}{h^3}\int \limits _{D_j}n_0(z)dz, \end{aligned}$$

where $\nu _j$ is an approximation of the average of the solution *n*(*t*, *z*) over $D_j$, $N_j$ is the set of indexes corresponding to the neighbours of $D_j$ and the coefficients $M_j$ and $B_{jl}$ are functions of *V*(*t*, *z*), $A(\theta )$, *r*(*z*) and *d*(*z*). A full detailed derivation of the scheme is given in Sect. 3. We can then introduce the following result involving the solution for this system:

### Theorem 2

For all non-negative $n_0\in L^p(D)$, $p>2$, there exists a unique non-negative solution for problem (5–6). Furthermore, the function ${\tilde{n}}_h(t,z)$ defined by

$$\begin{aligned} {\tilde{n}}_h(t,z)=\sum \limits _j\nu _j(t)\mathbb {1}_{D_j\cap D}, \end{aligned}$$

converges in $L^2(D_T)$ to the unique non-negative weak solution of (1–3) as *h* goes to zero.

The existence and non-negativity of the solution for (5–6) results from the Cauchy-Lipschitz theorem, while the convergence of ${\tilde{n}}_h(t,z)$ is the consequence of the compactness of the sequence.

The existence result in Theorem 1 will be treated in Sect. 3, but the uniqueness can be directly obtained from the variational formulation with the help of some *a posteriori* estimates. Let us proceed then to prove the uniqueness before addressing the existence. Assume that there exists a non-negative weak solution *n* for (1–3). Assume as well that there exist $0\leqslant t_0<t_1$ such that $\rho (t)\leqslant \max \{\rho (0),\frac{r^+}{d^-}\}$ for $t\in [0,t_0]$, and $\rho (t)>\max \{\rho (0),\frac{r^+}{d^-}\}$ for $t\in (t_0,t_1)$. We may take on the variational formulation $\varphi (t,z)=\chi _{\varepsilon }(t)$, where $\chi _{\varepsilon }(t)$ is a sequence satisfying $\chi _{\varepsilon }(t)\rightarrow \mathbb {1}_{t>t_0}(t)$ and $\chi '_{\varepsilon }(t)\rightarrow \delta _{t_0}(t)$. For example, we could consider $\chi _{\varepsilon }(t)=\frac{1}{2}(1-\cos (\frac{\pi (t-t_0+\varepsilon )}{2\varepsilon }))\mathbb {1}_{t\in [t_0,t_0+\varepsilon ]}+\mathbb {1}_{t>t_0+\varepsilon }$. This leads to the equality

$$\begin{aligned} (n(t),\chi _{\varepsilon }(t))_H=\int _0^t\int \limits _{D}(r(z)-d(z)\rho (t))n(t,z)dz\chi _{\varepsilon }(s) +\langle \chi '_{\varepsilon }(s),n(s)\rangle \Big )ds. \end{aligned}$$

Taking the limit when $\varepsilon$ goes to 0, we obtain that, for all $t>t_0$

$$\begin{aligned} \rho (t)=\rho (t_0)+\int _{t_0}^{t}\int \limits _{D}(r(z)-d(z)\rho (s))n(s,z)dzds, \end{aligned}$$

where $\rho (t)=\int \limits _{D}n(t,z)dz$ is a continuous function due to the fact that $n(t,z)\in {\mathcal {C}}([0,T],H)$. We can write the previous relation as

$$\begin{aligned} \rho (t)=\rho (t_0)+\int _{t_0}^{t}\int \limits _{D}d(z)\left( \frac{r(z)}{d(z)}-\frac{r^+}{d^-}+\frac{r^+}{d^-}-\rho (s)\right) n(s,z)dzds, \end{aligned}$$

which, thanks to the hypothesis on $\rho (t)$ over $(t_0,t_1)$, leads to

$$\begin{aligned} \rho (t)\leqslant \rho (t_0)\leqslant \max \left\{ \rho (0),\frac{r^+}{d^-}\right\} \end{aligned}$$

for all $t\in (t_0,t_1)$. This represents a contradiction, and implies that

7 $$\begin{aligned} \rho (t)\leqslant \max \left\{ \rho (0),\frac{r^+}{d^-}\right\} \end{aligned}$$

for all values of *t*.

Taking now $\varphi =n$ on the variational formulation, using standard arguments to bound the linear terms from *Q*[*n*] and the estimate (7) for the non-linear part together with the Gronwall Lemma, we obtain the relation

$$\begin{aligned} \frac{1}{2}\Vert n(t)\Vert ^2_H\leqslant \frac{1}{2}\Vert n(0)\Vert ^2_H+a\int \limits _0^t\frac{1}{2}\Vert n(s)\Vert ^2_Hds, \end{aligned}$$

for some real positive number *a*. Using Gronwall’s lemma, this relation implies that

$$\begin{aligned} \Vert n(t)\Vert ^2_H\leqslant \Vert n_0\Vert ^2_He^{2at}, \end{aligned}$$

for all values of *t*. Finally assume the existence of two non-negative weak solutions $n_1$ and $n_2$ for the same initial data $n_0$. Taking the difference between their respective variational formulations, choosing $\varphi =n_1-n_2$, we get the equality

$$\begin{aligned} \frac{1}{2}\Vert n_1-n_2\Vert ^2_H= \int \limits _0^t \langle Q [n_1]-Q [n_2],n_1-n_2\rangle ds. \end{aligned}$$

Once again, the linear part of $Q [n_1]-Q [n_2]$ can be easily bounded using standard methods, leading to

$$\begin{aligned} \frac{1}{2}\Vert n_1-n_2\Vert ^2_H\leqslant&a\int \limits _0^t\Vert n_1-n_2\Vert ^2_Hds-\int \limits _0^t\rho _1(s)\int \limits _{D}d(z)(n_1-n_2)^2dzds\\&-\int \limits _0^t(\rho _1(t)-\rho _2(t))\int \limits _Dn_2d(z)(n_1-n_2)dzds\\ \leqslant&\int \limits _0^t(a+d^+\Vert n_0\Vert _He^{as})\Vert n_1-n_2\Vert ^2_Hds \end{aligned}$$

Consequently, thanks to Gronwall’s lemma, $\Vert n_1-n_2\Vert ^2_H=0$ for all *t*, therefore, the solution is unique.

As stated before, the proof of existence of a weak solution will be carried on in Sect. 3. Following the ideas from Carrillo et al. (2020), the semi-discrete numerical scheme (5–6) is developed and the convergence of its solution to the solution of (1–3) is demonstrated. A fully discrete numerical scheme is obtained starting from the semi-discrete one and its convergence is also proved. Section 4 is devoted to the numerical simulations, starting with some numerical computations of the approximation error by comparing the results with an exact solution. Finally, the solutions of some examples with biological meaning is presented.

## Existence of a weak solution and numerical approximation

We aim to use the Finite Volume method in order to find a sequence of problems whose solutions converge to the solution of (4).

### Preliminaries on the finite volume method

Consider $h=1/M$ where *M* is a natural number and define the mesh

$$\begin{aligned} C_{ijk}=[\frac{i}{M},\frac{i+1}{M}]\times [\frac{j}{M},\frac{j+1}{M}]\times [\frac{k}{M},\frac{k+1}{M}], \end{aligned}$$

with $i,j,k=0,\dots ,M-1$, such that $\bigcup \limits _{i,j,k}C_{ijk}=[0,1]^3$.

Now introduce the sets

$$\begin{aligned} {\mathcal {M}}=\{C_{ijk}: C_{ijk}\cap D\ne \emptyset \} \end{aligned}$$

and

$$\begin{aligned} D_h=\bigcup \limits _{{\mathcal {M}}}C_{ijk}. \end{aligned}$$

For simplicity, we define $N:=|{\mathcal {M}}|$ as the amount of elements in ${\mathcal {M}}$, and denote each of its elements as $D_j$, for $j=1,\ldots ,N$. For each $D_j$, we denote its centre of mass as $\mathbf {z_j}:=(x_j,y_j,\theta _j)$. For each *j*, define $N_j$ as the set of indexes *l* such that $D_l$ and $D_j$ share a common boundary. Denote such common boundary as $\Gamma _{jl}$, its centre of mass as $\mathbf {z_{jl}}$ and ${\mathbf {n}}_{jl}$ the outer normal vector of $D_j$, in the direction of $D_l$. We remark that the distance between the centres of two neighbouring cells $D_j$ and $D_l$ will be equal to $|\mathbf {z_l}-\mathbf {z_j}|$. Having cubes as the mesh cells guarantees that the condition

8 $$\begin{aligned} {\mathbf {n}}_{jl}=\frac{\mathbf {z_l}-\mathbf {z_j}}{|\mathbf {z_l}-\mathbf {z_j}|}, \end{aligned}$$

is fulfilled.

It is important to remark that if *D* has a regular enough boundary (for example: smooth or polygonal), then the area of $D_h\setminus D$, which we denote as $|D_h\setminus D|$, will converge to zero as *h* vanishes. The approximated problem (1–3) is then given by

9 $$\begin{aligned} \partial _t {\tilde{n}}_h+\nabla \cdot \Big (V{\tilde{n}}_h-A(\theta )\nabla {\tilde{n}}_h\Big )&=(r(z)-d(z){\tilde{\rho }}_h(t)){\tilde{n}}_h, \text{ in } D_h \end{aligned}$$

10 $$\begin{aligned} \Big (V{\tilde{n}}_h-A(\theta )\nabla {\tilde{n}}_h\Big )\cdot {\mathbf {n}}&=0, \text{ for } \text{ all } z\in \partial D_h, \end{aligned}$$

11 $$\begin{aligned} {\tilde{n}}_h(0,z)&=n_0(z), \text{ for } \text{ all } z\in D_h, \end{aligned}$$

where ${\tilde{\rho }}_h(t)=\int \limits _{D_h} {\tilde{n}}_h(t,z)dz$. We propose a classical finite volume method based on local averages of the unknown density over cell grids defined by

12 $$\begin{aligned} \nu _{j}(t):=\frac{1}{h^3}\int \limits _{D_j}{\tilde{n}}_h(t,z)dz=n(t,\mathbf {\mathbf {z_j}})+{\mathcal {O}}(h^2). \end{aligned}$$

Assume that the coefficients and the solution from Eq. (9) are smooth. Then, integrating it over a cell $D_j$ yields the equality

$$\begin{aligned} \frac{d}{dt}\int \limits _{D_j}{\tilde{n}}_hdz=-\int \limits _{D_j}\nabla \cdot \Big (V{\tilde{n}}_h-A\nabla {\tilde{n}}_h\Big )dz+\int \limits _{D_j}r(z){\tilde{n}}_hdz-{\tilde{\rho }}_h(t)\int \limits _{D_j}d(z){\tilde{n}}_hdz. \end{aligned}$$

After integrating by parts and using the boundary conditions (10), we get

$$\begin{aligned} -\int \limits _{D_j}\nabla \cdot \Big (V{\tilde{n}}_h-A\nabla {\tilde{n}}_h\Big )dz=-\sum \limits _{l\in N_j}\int \limits _{\Gamma _{jl}}\Big (V{\tilde{n}}_h-A\nabla {\tilde{n}}_h\Big )\cdot {\mathbf {n}}_{jl}dS.\nonumber \end{aligned}$$

For a real function *f*(*t*), define the positive and negative part of *f* as

$$\begin{aligned} f^+(t)=\left\{ \begin{matrix} f(t),&{} \text{ if } &{} f(t)\geqslant 0,\\ &{}&{}\\ 0,&{} \text{ if } &{} f(t)<0, \end{matrix} \right. \end{aligned}$$

and

$$\begin{aligned} f^-(t)=\left\{ \begin{matrix} 0,&{} \text{ if } &{} f(t)\geqslant 0,\\ &{}&{}\\ f(t),&{} \text{ if } &{} f(t)<0, \end{matrix} \right. \end{aligned}$$

respectively. Using an upwind approximation technique for the advection term, we conclude

13 $$\begin{aligned} \int \limits _{\Gamma _{jl}}V{\tilde{n}}_h \cdot {\mathbf {n}}_{jl}dS=|\Gamma _{jl}|\Big (\nu _j(t)u^+_{jl}(t)+\nu _l(t)u^-_{jl}(t)\Big )+{\mathcal {O}}(h^{2}), \end{aligned}$$

where $u_{jl}(t)=V(t,\mathbf {z_{jl}})\cdot \vec {{\mathbf {n}}}_{jl}$. On the other hand, we have

$$\begin{aligned} -\int \limits _{\Gamma _{jl}}\Big (A\nabla {\tilde{n}}_h\Big )\cdot {\mathbf {n}}_{jl}dS=-|\Gamma _{jl}|\Big (A(\theta _{jl})\nabla {\tilde{n}}_h(\mathbf {z_{jl}})\Big )\cdot {\mathbf {n}}_{jl}=-|\Gamma _{jl}|\nabla {\tilde{n}}_h(\mathbf {z_{jl}})\cdot A(\theta _{jl}){\mathbf {n}}_{jl}.\nonumber \end{aligned}$$

As $A(\theta )$ is a diagonal matrix and ${\mathbf {n}}_{jl}$ is either one of the vectors from the euclidean canonical base, or one of their opposites, we have the relation

$$\begin{aligned} A(\theta _{jl}){\mathbf {n}}_{jl}=A_{jl}{\mathbf {n}}_{jl}, \end{aligned}$$

where $A_{jl}=(A(\theta _{jl}){\mathbf {n}}_{jl}\cdot {\mathbf {n}}_{jl})$. Notice that, thanks to hypothesis *(H3)*, there exists $\alpha >0$ such that $A_{jl}\geqslant \alpha$, for all *j* and *l*. So that, together with the expression of the normal vectors, (8) this implies

$$\begin{aligned} -\int \limits _{\Gamma _{jl}}\Big (A\nabla {\tilde{n}}_h\Big )\cdot {\mathbf {n}}_{jl}dS=-\frac{A_{jl}|\Gamma _{jl}|}{|\mathbf {z_l}-\mathbf {z_j}|}\Big (\nabla {\tilde{n}}_h(\mathbf {z_{jl}})\Big )\cdot \Big (\mathbf {z_l}-\mathbf {z_j}\Big ).\nonumber \end{aligned}$$

Due to the approximation of the gradient

$$\begin{aligned}\Big (\nabla {\tilde{n}}_h(\mathbf {z_{jl}})\Big )\cdot \Big (\mathbf {z_j}-\mathbf {z_l}\Big )={\tilde{n}}_h(\mathbf {z_l})-{\tilde{n}}_h(\mathbf {z_j})+{\mathcal {O}}(h^{2}), \end{aligned}$$

we can finally write

14 $$\begin{aligned} -\int \limits _{\Gamma _{jl}}\Big (A\nabla {\tilde{n}}_h\Big )\cdot {\mathbf {n}}_{jl}dS&=-\frac{A_{jl}|\Gamma _{jl}|}{|\mathbf {z_l}-\mathbf {z_j}|}\Big ({\tilde{n}}_h(\mathbf {z_j})-{\tilde{n}}_h(\mathbf {z_l}) \Big )+{\mathcal {O}}(h^{2})\nonumber \\&=-\frac{A_{jl}|\Gamma _{jl}|}{|\mathbf {z_l}-\mathbf {z_j}|}\Big (\nu _l(t)-\nu _j(t) \Big )+{\mathcal {O}}(h^{2}). \end{aligned}$$

Taking into account that

$$\begin{aligned} \rho _h(t)=\int \limits _{D_h}{\tilde{n}}_h(t,z)dz=\sum \limits _{l=1}^N\int \limits _{D_l}{\tilde{n}}_h(t,z)dz=\sum \limits _{l=1}^N h^3\nu _l(t), \end{aligned}$$

the reaction terms can be easily approximated

15 $$\begin{aligned} \int \limits _{D_j}r(z){\tilde{n}}_hdz-{\tilde{\rho }}_h(t)\int \limits _{D_j}d(z){\tilde{n}}_h dz&=h^3\Big (r(\mathbf {z_j})-\rho _h(t)d(\mathbf {z_j})\Big ){\tilde{n}}_h(t,\mathbf {z_j})+{\mathcal {O}}(h^5)\nonumber \\&=h^3\Big (r_j-{\tilde{\rho }}_h(t)d_j\Big ) \nu _j(t)+{\mathcal {O}}(h^5)\nonumber \\&=h^3\Big (r_j-d_j\sum \limits _{l=1}^N h^3\nu _l(t)\Big ) \nu _j(t)+{\mathcal {O}}(h^5), \end{aligned}$$

where we have adopted the notation $r_j:=r(\mathbf {z_j})$ and $d_j:=d(\mathbf {z_j})$. Finally, using again (12), we get

$$\begin{aligned} \frac{d}{dt}\int \limits _{D_j} {\tilde{n}}_h(z)dz=h^3\nu '_j(t). \end{aligned}$$

Consequently, collecting (13), (14) and (15), and getting rid of the approximation orders we obtain the semi-discrete scheme

16 $$\begin{aligned} \frac{d}{dt}\nu _j(t)=M_{j}(t,{\tilde{\rho }}_h(t))\nu _j(t)+\sum \limits _{l\in N_j}B_{jl}(t)\nu _l(t), \end{aligned}$$

where

$$\begin{aligned} M_j(t,{\tilde{\rho }}_h(t))&= -\sum \limits _{l\in N_j}\frac{|\Gamma _{jl}|}{h^3}\Big (u^+_{jl}(t)+\frac{A_{jl}}{|\mathbf {z_l}-\mathbf {z_j}|}\Big )+\Big (r_j-d_j{\tilde{\rho }}_h(t)\Big ),\\ B_{jl}&=\frac{|\Gamma _{jl}|}{h^3}\Big (-u^-_{jl}(t)+\frac{A_{jl}}{|\mathbf {z_l}-\mathbf {z_j}|}\Big ). \end{aligned}$$

This system of equations can be complemented with the set of initial data

17 $$\begin{aligned} \nu _j(0)=\nu ^0_{j}:=\frac{1}{h^3}\int _{D_j}{\overline{n}}_0(z)dz, \end{aligned}$$

where ${\overline{n}}_0(z)$ is the extension by 0 of $n_0(z)$ to all of ${\mathbb {R}}^3$.

### Global existence, uniqueness, positivity and boundedness of the solution for the semi-discrete scheme

We prove the local existence and uniqueness of the solution for the problem (16–17), by using the Cauchy-Lipschitz theorem. Then, such solution is proved to be non-negative and, as a consequence, bounded independently of *t*. Finally the boundedness property is used to prove the global existence of the solution.

#### Proposition 3

(Local existence of solution) For all sets of initial data $$\{\nu ^0_{j}\}$$, there exists $$0<T^*<\infty $$ such that the problem (16–17) has an unique solution over $$[0,T^*)$$. Furthermore, if $$\nu _{j}^0\geqslant 0$$ for all *j*, then $$\nu _j(t)\geqslant 0$$ for all time $$t\in [0,T^*)$$ and all *j*.

#### Proof

The RHS term in (16) is Lipschitz continuous for all values of *t* and $$\nu _j$$, therefore the existence and uniqueness of solution over a certain interval $$[0,T^*)$$ is a direct consequence of the Cauchy-Lipschitz theorem.

On the other hand, consider a strictly positive set of initial values $\nu _{j}^0$ and define the continuous function $f(t)=\min \limits _{j}\nu _{j}(t)$. If $f(t)\geqslant 0$ for all $t<T^*$, then the solution remains positive at all times. If $f(t)<0$ for some $t\in (0,T^*)$, then there exists $t_0>0$ such that $f(t_0)=0$ and $f(t)\geqslant 0$ for $t<t_0$. This implies the existence of $j_0$ such that $\nu _{j_0}(t_0)=0$ with $\nu '_{j_0}(t_0)\leqslant 0$. If $\nu _l(t_0)>0$ for some $l\in N_{j_0}$, then, thanks to (16) we have

$$\begin{aligned} \nu '_{j_0}(t_0)&=\sum \limits _{l\in N_j}B_{jl}(t_0)\nu _l(t_0)\\&=\sum \limits _{l\in N_j}\frac{|\Gamma _{jl}|}{h^3}\Big (-u^-_{jl}(t_0)+\frac{A_{jl}}{|\mathbf {z_l}-\mathbf {z_j}|}\Big )\nu _l(t_0)>0, \end{aligned}$$

which is a contradiction with the previously established fact that $\nu '_{j_0}(t_0)\leqslant 0$. Consequently $\nu _l(t_0)=0$ for all $l\in N_{j_0}$. Furthermore, from the definitions of *f*(*t*) and $t_0$, we also have that $\nu '_l(t_0)\leqslant 0$ for all $l\in N_{j_0}$. We can iterate the previous argument in order to obtain that $\nu _j(t_0)=0$ for all *j* and consequently, thanks to the uniqueness of the solution, $\nu _j(t)\equiv 0$ for all *j* and all *t*. This is a contradiction with the assumption that *f*(*t*) is negative for some value of *t* and consequently $f(t)\geqslant 0$ for all *t*.

Finally, for non-negative initial values $\nu _j^0$ and $\varepsilon$ small enough, we define $\nu _j^{\varepsilon }$ as

$$\begin{aligned} \nu _j^{\varepsilon }= \left\{ \begin{matrix} \nu _j^{0}&{} \text{ if } \nu _j^{0}>0,\\ &{}\\ \varepsilon &{} \text{ if } \nu _j^{0}=0. \end{matrix} \right. \end{aligned}$$

Thanks to the previous step, the solution of (16) associated to $\nu _j^{\varepsilon }$ remains non-negative for all *t* and all $\varepsilon$. Hence, thanks to the continuous dependence of the solution of a system with respect to its initial data, we conclude that $\nu _{j}^0\geqslant 0$ implies $\nu _j(t)\geqslant 0$ for all $t\in [0,T^*)$. $\square$

#### Proposition 4

(Discrete $L^1$ bound) The $L^1$ norm ${\tilde{\rho }}_h(t):=\sum \limits _lh^3\nu _l(t)$ satisfies the bounds

18 $$\begin{aligned} {\underline{\rho }}:=\min \{{\tilde{\rho }}_h(0),\frac{r^-}{d^+}\}\leqslant {\tilde{\rho }}_h(t)\leqslant \max \{{\tilde{\rho }}_h(0),\frac{r^+}{d^-}\}=:{\overline{\rho }}, \forall t\geqslant 0, \end{aligned}$$

where $r^-$, $r^+$, $d^-$ and $d^+$ are the bounds given in *(H1)* for *r*(*z*) and *d*(*z*) respectively.

#### Proof

Multiplying (16) by $h^3$ for each *j*, adding up all the equations, recalling that $|\Gamma _{jl}|=|\Gamma _{lj}|$, $u^+_{jl}=-u^-_{lj}$, $A_{jl}=A_{lj}$ and $|\mathbf {z_l}-\mathbf {z_j}|=|\mathbf {z_j}-\mathbf {z_l}|$ we obtain that

$$\begin{aligned} {\tilde{\rho }}'_h(t)=\sum \limits _{j=1}^N\Big (r_j-d_j{\tilde{\rho }}_h(t)\Big )h^3\nu _j(t).\nonumber \end{aligned}$$

The non-negativity of the solution implies then

$$\begin{aligned} \Big (r^--d^+{\tilde{\rho }}_h(t)\Big ){\tilde{\rho }}_h(t)\leqslant {\tilde{\rho }}'_h(t)\leqslant \Big (r^+-d^-{\tilde{\rho }}_h(t)\Big ){\tilde{\rho }}_h(t). \end{aligned}$$

These differential inequalities directly imply the bounds over ${\tilde{\rho }}_h(t)$. $\square$

Notice that the $L^1$ bounds are independent of *t*. Additionally, the upper bound also implies that

$$\begin{aligned} h^3\nu _j(t)\leqslant {\tilde{\rho }}_h(t)\leqslant {\overline{\rho }}, \text{ for } \text{ all } t\in [0,T^*), \text{ for } \text{ all } j, \end{aligned}$$

So that in general $\nu _j(t)\leqslant \frac{{\overline{\rho }}}{h^3}$, which is the key estimate in order to prove global existence of solution for (16–17).

#### Proposition 5

(Global existence of solution) For all sets of initial data $\{\nu ^0_{j}\}$, there exists a unique solution of problem (16–17) for all $t>0$. Such solution is non-negative and satisfies the estimate (18).

#### Proof

For each *h*, assume that there exists a finite maximal time of existence $$T_h$$. However, the estimate (18) on $$ {\tilde{\rho }}_h(t)$$ implies that for all *j*, $$\nu _j(T_h)\leqslant \frac{{\overline{\rho }}}{h^3}<\infty $$, which, thanks to the Lipschitz continuity of the right hand side of (16) allows to extend to solution to a certain interval $$[T_h,T^*_h)$$, contradicting this way the maximality of $$T_h$$. $$\square $$

### Discrete gradient, $L^2$ norm estimate and compactness result

In this section we introduce some piecewise constant functions depending on the solution of (16–17) together with some estimates related to such functions. Then, some compactness properties will be proved in order to ensure that such functions converge to some function that will be proved to be a weak solution of (1–2), and their derivatives, respectively.

We first introduce $n_h(t,z)$ defined as

$$\begin{aligned} n_h(t,z)=\sum \limits _{j=1}^N\nu _j(t)\mathbb {1}_{D_j}(z). \end{aligned}$$

Notice that $\Vert n_h\Vert _{L^1}={\tilde{\rho }}_h(t)$. Now, for each $l\in N_j$, define the polygonal subsets of $D_{j}$, denoted $D_{jl}$, having $\Gamma _{jl}$ as the common side and $\mathbf {z_j}$ as a vertex. The subsets $D_{jl}$ are pyramids of area $s_{jl}=\frac{|\Gamma _{jl}|d(\mathbf {z_j},\Gamma _{jl})}{3}=\frac{h^3}{6}$. Let us define the piecewise constant functionThis function can be regarded as a discrete gradient for $n_h(t)$.

#### Proposition 6

($L^2$ bound) For each value of *h*, define the space $H_h:=L^2(D_h)$. Then, there exists positive constants *a* and *b*, independent of *h*, such that the functions $n_h(t,z)$ and $v_h(t,z)$ satisfy the following estimate

19 $$\begin{aligned} \Vert n_h\Vert ^2_{H_h}+a\int \limits _0^T\Vert v_h\Vert ^2_{H_h}\leqslant e^{bT}\Vert n_0\Vert ^2_{H_h}, \text{ for } \text{ all } T>0. \end{aligned}$$

#### Proof

Multiplying Eq. (16) by $h^3\nu _j(t)$ for each *j*, and adding them up, we obtain the relation

20 $$\begin{aligned} \sum \limits _{j=1}^Nh^3\nu _j(t)\nu '_j(t)=A(t)+D(t)+R(t), \end{aligned}$$

where

$$\begin{aligned} A(t)&=-\sum \limits _{j=1}^N\nu _j(t)\sum \limits _{l\in N_j}|\Gamma _{jl}|\Big (\nu _j(t)u^+_{jl}(t)+\nu _l(t)u^-_{jl}(t)\Big ),\\ D(t)&=\sum \limits _{j=1}^N\nu _j(t)\sum \limits _{l\in N_j}\frac{A_{jl}|\Gamma _{jl}|}{|\mathbf {z_l}-\mathbf {z_j}|}\Big (\nu _l(t)-\nu _j(t) \Big ),\\ R(t)&=\sum \limits _{j=1}^Nh^3\Big (r_j-d_j\sum \limits _l h^3\nu _l(t)\Big ) \nu ^2_j(t). \end{aligned}$$

Let us proceed to estimate each of these terms. In order to simplify the notation, we define

$$\begin{aligned} \mu _{jl}(t)=|\Gamma _{jl}|(\nu _j(t)u^+_{jl}(t)+\nu _l(t)u^-_{jl}(t)), \end{aligned}$$

and write

$$\begin{aligned} A(t)=-\frac{1}{2}\sum \limits _{j=1}^N\sum \limits _{l\in N_j}\Big (\nu _j(t)\mu _{jl}(t)+\nu _l(t)\mu _{lj}(t)\Big ). \end{aligned}$$

Consequently, knowing that $-u^+_{jl}=u^-_{lj}\leqslant 0$, we have

$$\begin{aligned} A(t)&=\frac{1}{2}\sum \limits _{j=1}^N\sum \limits _{l\in N_j}|\Gamma _{jl}|\Big (\nu _l(t)-\nu _j(t)\Big )\Big (u^+_{jl}(t)\nu _j(t)+u^-_{jl}(t)\nu _l(t)\Big )\\&=\frac{1}{2}\sum \limits _{j=1}^N\sum \limits _{l\in N_j}|\Gamma _{jl}|\Big (\nu _l(t)-\nu _j(t)\Big )\Big (u_{jl}(t)\nu _j(t)+u^-_{jl}(t)(\nu _l(t)-\nu _j(t))\Big )\\&\leqslant \frac{1}{2}\sum \limits _{j=1}^N\sum \limits _{l\in N_j}|\Gamma _{jl}|\Big (\nu _l(t)-\nu _j(t)\Big )\Big (u_{jl}(t)\nu _j(t)\Big )\\&= \sum \limits _{j=1}^N\sum \limits _{l\in N_j}\frac{|\Gamma _{jl}|}{|\mathbf {z_l}-\mathbf {z_j}|}|\mathbf {z_{jl}}-\mathbf {z_j}|\Big (\nu _l(t)-\nu _j(t)\Big )\Big (u_{jl}(t)\nu _j(t)\Big )\,, \end{aligned}$$

where we used that $|\mathbf {z_l}-\mathbf {z_j}|=2|\mathbf {z_{jl}}-\mathbf {z_j}|$. From the definition of $u_{jl}$, we conclude that

$$\begin{aligned} |u_{jl}|\leqslant |V(t,\mathbf {z_{jl}})\cdot \vec {{\mathbf {n}}}_{jl} |\leqslant {\overline{V}}, \end{aligned}$$

where ${\overline{V}}:=\max \limits _{z,t}|V(t,z)|$. This implies

$$\begin{aligned} |A(t)|&\leqslant {\overline{V}}\sum \limits _{j=1}^N\sum \limits _{l\in N_j}\frac{|\Gamma _{jl}|}{|\mathbf {z_l}-\mathbf {z_j}|}|\mathbf {z_{jl}}-\mathbf {z_j}|\Big (|\nu _l(t)-\nu _j(t)|\Big )\nu _j(t)\\&={\overline{V}}\sum \limits _{j=1}^N\sum \limits _{l\in N_j}\frac{|\Gamma _{jl}|}{|\mathbf {z_l}-\mathbf {z_j}|}|\mathbf {z_{jl}}-\mathbf {z_j}|\frac{\Big (|\nu _l(t)-\nu _j(t)|\Big )}{s_{jl}}\nu _j(t)s_{jl}\\&={\overline{V}}\int \limits _{D}|v_h(t,z)||n_h(t,z)|dz. \end{aligned}$$

Then, for all $\varepsilon >0$, Young’s inequality implies

21 $$\begin{aligned} |A(t)|\leqslant {\overline{V}}\Big (\frac{\varepsilon }{2} \Vert v_h\Vert ^2_{H_h}+2\varepsilon ^{-1}\Vert n_h\Vert ^2_{H_h}\Big ). \end{aligned}$$

On the other hand

$$\begin{aligned} D(t)&=\frac{1}{2}\sum \limits _{j=1}^N\sum \limits _{l\in N_j}\frac{A_{jl}|\Gamma _{jl}|}{|\mathbf {z_l}-\mathbf {z_j}|}\Big (\nu _j(t)\Big (\nu _l(t)-\nu _j(t) \Big )+\nu _l(t)\Big (\nu _j(t)-\nu _l(t) \Big )\Big )\\&=-\frac{1}{2}\sum \limits _{j=1}^N\sum \limits _{l\in N_j}\frac{A_{jl}|\Gamma _{jl}|}{|\mathbf {z_l}-\mathbf {z_j}|}\Big (\nu _l(t)-\nu _j(t)\Big )^2\\&=-\frac{1}{2}\sum \limits _{j=1}^N\sum \limits _{l\in N_j}\frac{A_{jl}s_{jl}|\mathbf {z_l}-\mathbf {z_j}|}{|\Gamma _{jl}||\mathbf {z_{jl}}-\mathbf {z_j}|^2}\frac{|\Gamma _{jl}|^2|\mathbf {z_{jl}}-\mathbf {z_j}|^2}{d^2_{jl}}\frac{\Big (\nu _l(t)-\nu _j(t)\Big )^2}{s^2_{jl}}s_{jl}. \end{aligned}$$

The ellipticity and boundedness of the matrix $A(\theta )$ imply that there exist positive constants ${\underline{\alpha }}$, ${\overline{\alpha }}$ such that ${\underline{\alpha }}\leqslant A_{jl}\leqslant {\overline{\alpha }}$ for all *j* and *l*. From this and the value of $s_{jl}$ we deduce that

$$\begin{aligned} \frac{A_{jl}s_{jl}|\mathbf {z_l}-\mathbf {z_j}|}{|\Gamma _{jl}||\mathbf {z_{jl}}-\mathbf {z_j}|^2}=\frac{4A_{jl}h^4}{6h^4}\geqslant \frac{2{\underline{\alpha }} }{3}, \end{aligned}$$

and consequently

22 $$\begin{aligned} D(t)&\leqslant -\frac{{\underline{\alpha }} }{3}\sum \limits _{j=1}^N\sum \limits _{l\in N_j}\frac{|\Gamma _{jl}|^2|\mathbf {z_{jl}}-\mathbf {z_j}|^2}{d^2_{jl}}\frac{\Big (\nu _l(t)-\nu _j(t)\Big )^2}{s^2_{jl}}s_{jl}=-\frac{{\underline{\alpha }} }{3}\Vert v_h\Vert ^2_{H_h}, \end{aligned}$$

notice that the last identity is true since $v_h$ is a piecewise constant function. Using the bounds over *r*(*z*) and the positiveness of *d*(*z*) and ${\tilde{\rho }}_h(t)$ we see that

23 $$\begin{aligned} R(t)&:=\sum \limits _{j=1}^Nh^3\Big (r_j-d_j\sum \limits _l h^3\nu _l(t)\Big ) \nu ^2_j(t) = \sum \limits _{j=1}^Nh^3\Big (r_j-d_j{\tilde{\rho }}_h(t)\Big ) \nu ^2_j(t)\nonumber \\&\leqslant r^+\sum \limits _{j=1}^Nh^3\nu ^2_j(t)=r^+\Vert n_h\Vert ^2_{H_h}. \end{aligned}$$

Using (21), (22) and (23) in (20), with the relation

$$\begin{aligned} \sum \limits _{j=1}^Nh^3\nu _j(t)\nu '_j(t)=\frac{1}{2}\Big (\sum \limits _{j=1}^Nh^3\nu ^2_j(t)\Big )'=\frac{1}{2}\Big (\Vert n_h\Vert ^2_{H_h}\Big )', \end{aligned}$$

yields the differential inequality

24 $$\begin{aligned} \frac{1}{2}\Big (\Vert n_h\Vert ^2_{H_h}\Big )'+\Big (\frac{{\underline{\alpha }} }{3}-\frac{\varepsilon {\overline{V}}}{2}\Big )\Vert v_h\Vert ^2_{H_h}\leqslant \Big (\frac{\varepsilon ^{-1}{\overline{V}}}{2}+r^+\Big )\Vert n_h\Vert ^2_{H_h}, \end{aligned}$$

which, after taking $\varepsilon =\frac{{\underline{\alpha }} }{3{\overline{V}}}$ and using Gronwall’s lemma, leads to the estimate (19). $\square$

The result of Proposition 6 can be easily generalised if instead of multiplying each Eq. (16) by $h^3\nu _j(t)$, we multiply by $h^3\nu ^{p-1}_j(t)$, for any $p>1$, which would lead to the following uniform $L^p$ bound:

#### Proposition 7

($L^p$ bound) There exists positive constants $a_p$ and $b_p$, independents of *h*, such that the functions $n^p_h(t,z)$ and $v^p_h(t,z)$ defined assatisfy the following estimate

25 $$\begin{aligned} \Vert n^{p}_h\Vert ^2_{H_h}+a_p\int \limits _0^T\Vert v^{p}_h\Vert ^2_{H_h}\leqslant e^{b_pT}\Vert n_0\Vert ^2_{L^p(D)}, \text{ for } \text{ all } T>0. \end{aligned}$$

Before stating the compactness result that will allow us to extract a convergent subsequence from $n_h$ we give two important results that are a consequence from estimate (19).

#### Proposition 8

For all vectors $\eta \in {\mathbb {R}}^3$ define the translation operator $\pi _{\eta }:L^2({\mathbb {R}}^3)\rightarrow L^2({\mathbb {R}}^3)$ as $\pi _{\eta }(u)(z)=u(z+\eta )$. Then

$$\begin{aligned} \lim \limits _{\eta \rightarrow 0}\Vert \pi _{\eta }{\overline{n}}_h-{\overline{n}}_h\Vert _{L^2({\mathbb {R}}^3\times (0,T))}=0, \end{aligned}$$

uniformly in *h*, where ${\overline{n}}_h$ represents the extension by 0 of $n_h$ to all of ${\mathbb {R}}^3$.

#### Proof

The proof of this proposition follows the same steps as Lemma 18.3 and Remark 18.8 in Eymard et al. (2000). In particular, thanks to the discrete trace inequality given in the Lemma 10.5 within the same reference, we can prove the existence of a constant *C*, independent of *h* and $\eta$, such that

$$\begin{aligned} \Vert \pi _{\eta }{\overline{n}}_h-{\overline{n}}_h\Vert ^2_{L^2({\mathbb {R}}^3\times (0,T))}\leqslant |\eta |C\left( \int \limits _0^T\left( \Vert v_h\Vert ^2_{H_h}+\Vert n_h\Vert ^2_{H_h}\right) dt\right) , \end{aligned}$$

and we get the result from Proposition 8 thanks to (19). $\square$

From now on we introduce the notation $D_T:=(0,T)\times D$.

#### Proposition 9

There exists a constant $C_1$, independent of *h*, such that for all $\psi \in {\mathcal {D}}(D_T)$,

$$\begin{aligned} \left|\int \limits _0^T\langle \frac{d {\overline{n}}_h}{dt},\psi \rangle _{H^{-3}\times H^{3}}dt\right|\leqslant C_1\sum \limits _{|k|\leqslant 3}\Vert \partial ^k_{z}\psi \Vert _{L^2(D_T)}. \end{aligned}$$

#### Proof

Using the scheme (16), we have that, for all $\psi \in {\mathcal {D}}(D)$

$$\begin{aligned} \langle \frac{d {\overline{n}}_h}{dt},\psi \rangle _{H^{-3}\times H^{3}}&=\sum _j\frac{d\nu _j(t)}{dt}\int \limits _{D_j}\psi dz\\&=\sum _jh^3\Big (M_{j}(t,{\tilde{\rho }}_h(t))\nu _j(t)+\sum \limits _{l\in N_j}B_{jl}(t)\nu _l(t)\Big )\psi _j, \end{aligned}$$

where $\psi _j=h^{-3}\int \limits _{D_j}\psi dz$ is the mean value of $\psi$ over $D_j$. Reordering the terms in this equality we have

$$\begin{aligned} \langle \frac{d{\overline{n}}_h}{dt},\psi \rangle _{H^{-3}\times H^{3}}=&\frac{1}{2}\sum \limits _{j=1}^N\sum \limits _{l\in N_j}|\Gamma _{jl}|\Big (\psi _l-\psi _j\Big )\Big (u^+_{jl}(t)\nu _j(t)+u^-_{jl}(t)\nu _l(t)\Big )\\&-\frac{1}{2}\sum \limits _{j=1}^N\sum \limits _{l\in N_j}\frac{A_{jl}|\Gamma _{jl}|}{|\mathbf {z_l}-\mathbf {z_j}|}\Big (\nu _l(t)-\nu _j(t) \Big )\Big (\psi _l-\psi _j\Big )\\&+\sum \limits _{j=1}^Nh^3\Big (r_j-d_j{\tilde{\rho }}_h(t)\Big )\nu _j\psi _j \end{aligned}$$

We can bound each term on this equality as follows: From the definition of $u_{jl}(t)$ we get that $|u^{\pm }_{jl}|\leqslant \Vert V\Vert _{L^{\infty }(D_T)}$ and consequently

$$\begin{aligned}&\Big |\frac{1}{2}\sum \limits _{j=1}^N\sum \limits _{l\in N_j}|\Gamma _{jl}|\Big (\psi _l-\psi _j\Big )\Big (u^+_{jl}(t)\nu _j(t)+u^-_{jl}(t)\nu _l(t)\Big )\Big |\\&\quad =\Big |\frac{1}{2}\sum \limits _{j=1}^N\sum \limits _{l\in N_j}h^3\left( \frac{\psi _l-\psi _j}{h}\right) \Big (u^+_{jl}(t)\nu _j(t)+u^-_{jl}(t)\nu _l(t)\Big )\Big |\\&\quad \leqslant \frac{1}{2}\Vert \psi \Vert _{{\mathcal {C}}^1(D)}\Vert V\Vert _{L^{\infty }(D_T)}\sum \limits _{j=1}^N\sum \limits _{l\in N_j}h^3\Big (\nu _j(t)+\nu _l(t)\Big )\\&\quad \leqslant 3\Vert \psi \Vert _{{\mathcal {C}}^1(D)}\Vert V\Vert _{L^{\infty }(D_T)}\Vert n\Vert _{H_h}\,. \end{aligned}$$

Using the previously established relation $A_{jl}\leqslant {\overline{\alpha }}$ we get

$$\begin{aligned}&\Big |\frac{1}{2}\sum \limits _{j=1}^N\sum \limits _{l\in N_j}\frac{A_{jl}|\Gamma _{jl}|}{|\mathbf {z_l}-\mathbf {z_j}|}\Big (\nu _l(t)-\nu _j(t) \Big )\Big (\psi _l-\psi _j\Big )\Big |\\&\quad =\Big |\frac{1}{2}\sum \limits _{j=1}^N\sum \limits _{l\in N_j}A_{jl}h^3\Big (\frac{\nu _l(t)-\nu _j(t)}{h} \Big )\Big (\frac{\psi _l-\psi _j}{h}\Big )\Big |\\&\quad \leqslant {\overline{\alpha }}\Vert \psi \Vert _{{\mathcal {C}}^1(D)}\sum \limits _{j=1}^N\sum \limits _{l\in N_j}\frac{h^3}{2}\Big |\frac{\nu _l(t)-\nu _j(t)}{h} \Big |\\&\quad \leqslant {\overline{\alpha }}\Vert \psi \Vert _{{\mathcal {C}}^1(D)}\sum \limits _{j=1}^N\Vert v_h\Vert _{H_h}\,. \end{aligned}$$

Finally, using the boundedness of *r*(*z*), *d*(*z*) and ${\tilde{\rho }}_h$ we see that

$$\begin{aligned} \Big |\sum \limits _{j=1}^Nh^3\Big (r_j-d_j{\tilde{\rho }}_h(t)\Big )\nu _j\psi _j\Big |&\leqslant \sum \limits _{j=1}^Nh^3|r_j-d_j{\tilde{\rho }}_h(t)||\psi _j|\nu _j\\&\leqslant (r^++d^+{\overline{\rho }})\Vert \psi \Vert _{{\mathcal {C}}^1(D)}\sum \limits _{j=1}^Nh^3\nu _j\\&\leqslant (r^++d^+{\overline{\rho }})\Vert \psi \Vert _{{\mathcal {C}}^1(D)}\Vert n\Vert _{H_h}\,. \end{aligned}$$

Putting everything together, we obtain the existence of a constant *C* independent of *h*, such that

$$\begin{aligned} |\langle \frac{d {\overline{n}}}{dt},\psi \rangle _{H^{-3}\times H^{3}}|\leqslant C\Vert \psi \Vert _{{\mathcal {C}}^1(D)}\Big (\Vert n\Vert _{H_h}+\Vert v_h\Vert _{H_h}\Big ), \text{ for } \text{ all } t\in (0,T). \end{aligned}$$

Finally, using the inclusions $H^3(D)\subset {\mathcal {C}}^{1,\frac{1}{2}}(D)\subset {\mathcal {C}}^1(D)$ that hold true in any open subset of ${\mathbb {R}}^3$ with smooth enough boundary thanks to the Sobolev inequalities, integrating over (0, *T*), using the Cauchy-Schwartz inequality and estimate (19), we get to the result stated in the proposition. $\square$

#### Proposition 10

If $n_0\in L^p(D)$ for some $p>2$, then there exists a function $n\in L^2(0,T;{\mathcal {V}})$ such that, up to the extraction of a sub-sequence, the sequence of functions $n_h$ strongly converges to *n* in $L^2(D_T)$ and $v_h$ weakly converges to $\nabla n$ in $L^2(D_T)$.

#### Proof

Propositions 8 and 9 give all the necessary tools in order to ensure the relative compactness of the set of functions $\{n_h\}_h$ in $L^2(D_T)$. In order to prove that such set is indeed compact, we will use a variant adapted to our purposes of the proof of Moussa (2016, Theorem 3) to show that $\{n_h\}_h$ is a Cauchy sequence in $L^2(D_T)$. We consider a sequence of mollifiers $\Phi _{\varepsilon }(z)=\varepsilon ^{-3}\Phi (\varepsilon ^{-1}z)$ for a positive, symmetric function $\Phi \in {\mathcal {D}}(B(0,1))$ satisfying $\int _{{\mathbb {R}}^{3}}\Phi (z)dz=1$.

*Step 1:* We claim that

$$\begin{aligned} \lim \limits _{\varepsilon \rightarrow 0}\Vert \Phi _{\varepsilon }*{\overline{n}}_h-{\overline{n}}_h\Vert _{L^2((0,T)\times {\mathbb {R}}^3)}=0, \end{aligned}$$

uniformly on *h*.

We have

$$\begin{aligned} |\Phi _{\varepsilon }*{\overline{n}}_h(z)-{\overline{n}}_h(z)|&\leqslant \int \limits _{{\mathbb {R}}^3}|{\overline{n}}_h(z-y)-{\overline{n}}_h(z)|\Phi _{\varepsilon }(y)dy\\&\leqslant \left( \int \limits _{{\mathbb {R}}^3}|{\overline{n}}_h(z-y)-{\overline{n}}_h(z)|^2\Phi _{\varepsilon }(y)dy\right) ^{1/2}, \end{aligned}$$

thanks to the Cauchy-Schwarz inequality and the value of the integral of $\Phi _{\varepsilon }(y)$. Consequently

$$\begin{aligned} \Vert \Phi _{\varepsilon }*{\overline{n}}_h-{\overline{n}}_h\Vert _{L^2((0,T)\times {\mathbb {R}}^3 )}&\leqslant \int \limits _0^T\int \limits _{{\mathbb {R}}^3}\int \limits _{{\mathbb {R}}^3}|{\overline{n}}_h(z-y)-{\overline{n}}_h(z)|^2\Phi _{\varepsilon }(y)dydzdt\\&= \int \limits _{B(0,\varepsilon )}\Phi _{\varepsilon }(y)\Vert \pi _{-y}{\overline{n}}_h-{\overline{n}}_h\Vert ^2_{L^2((0,T)\times {\mathbb {R}}^3)}dy, \end{aligned}$$

and due to Proposition 8, we obtain the strong convergence of $\Phi _{\varepsilon }*{\overline{n}}_h$ to ${\overline{n}}_h$ in $L^2((0,T)\times {\mathbb {R}}^3)$, uniformly in *h*.

*Step 2:* We prove that for every fixed $\varepsilon$ and any compact $\omega \subset D$, the sequence $\Phi _{\varepsilon }*{\overline{n}}_h$ is uniformly bounded in $H^1((0,T)\times \omega )$.

Thanks to Young’s inequality, for a fixed $\varepsilon$,

$$\begin{aligned} \Vert \nabla (\Phi _{\varepsilon }*{\overline{n}}_h)\Vert _{L^2(D_T)}\leqslant \Vert \nabla \Phi _{\varepsilon }\Vert _{L^2(D)} \Vert {\overline{n}}_h \Vert _{L^1(D_T)} \leqslant C_{\varepsilon }{\overline{\rho }}T. \end{aligned}$$

Furthermore, for any compact $\omega \subset D$, $\psi \in {\mathcal {D}}((0,T)\times \omega )$ and $\varepsilon$ small enough, we have $\Phi _{\varepsilon }*\psi \in {\mathcal {D}}(D_T)$ and consequently, using Proposition 9 and Young’s inequality for the convolution product, we get

$$\begin{aligned} \left|\int \limits _0^T\langle \frac{d }{dt}\Phi _{\varepsilon }*{\overline{n}}_h,\psi \rangle dt\right|&\leqslant \int \limits _0^T\left|\langle \frac{d {\overline{n}}_h}{dt},\Phi _{\varepsilon }*\psi \rangle \right|dt\\&\leqslant C\sum \limits _{|k|\leqslant 3}\Vert \partial ^k_{z}\Phi _{\varepsilon }*\psi \Vert _{L^2(D_T)}\\&\leqslant C_{\varepsilon }\Vert \psi \Vert _{L^1(D_T)}. \end{aligned}$$

Then, by duality, for a fixed $\varepsilon$, $\frac{d }{dt}\Phi _{\varepsilon }*{\overline{n}}_h$ is uniformly bounded in $L^{\infty }((0,T)\times \omega )$ and we conclude that for each fixed $\varepsilon$, $\Phi _{\varepsilon }*{\overline{n}}_h$ is uniformly bounded in $H^1((0,T)\times \omega )$.

*Step 3:* We claim that, for all compacts $\omega \subset D$, the sequence ${\overline{n}}_{h}$ is a Cauchy sequence in $L^{2}((0,T)\times \omega )$.

Write

$$\begin{aligned} {\overline{n}}_{h_1}-{\overline{n}}_{h_2}=({\overline{n}}_{h_1}-\Phi _{\varepsilon }*{\overline{n}}_{h_1})+(\Phi _{\varepsilon }*{\overline{n}}_{h_1}-\Phi _{\varepsilon }*{\overline{n}}_{h_2})+(\Phi _{\varepsilon }*{\overline{n}}_{h_2}-{\overline{n}}_{h_2}). \end{aligned}$$

Thanks to *Step 1*, for all $\eta >0$, we can fix $\varepsilon$ in such a way that

$$\begin{aligned} \Vert {\overline{n}}_{h_1}-\Phi _{\varepsilon }*{\overline{n}}_{h_1}\Vert _{L^{2}((0,T)\times \omega )}<\eta /3,\\ \Vert \Phi _{\varepsilon }*{\overline{n}}_{h_2}-{\overline{n}}_{h_2}\Vert _{L^{2}((0,T)\times \omega )}<\eta /3. \end{aligned}$$

Thanks to *Step 2* and Rellich-Kondrachov’s theorem, we know that for a fixed $\varepsilon$, $\Phi _{\varepsilon }*{\overline{n}}_h$ is a Cauchy sequence in $L^{2}((0,T)\times \omega )$, hence, for $h_1$ and $h_2$ close enough

$$\begin{aligned} \Vert \Phi _{\varepsilon }*{\overline{n}}_{h_1}-\Phi _{\varepsilon }*{\overline{n}}_{h_2}\Vert _{L^{2}((0,T)\times \omega )}<\eta /3 \end{aligned}$$

Consequently, for all positive $\eta >0$, there exists $h_1$ and $h_2$ such that

$$\begin{aligned} \Vert {\overline{n}}_{h_1}-{\overline{n}}_{h_2}\Vert _{L^{2}((0,T)\times \omega )}<\eta , \end{aligned}$$

which proves that ${\overline{n}}_{h}$ is a Cauchy sequence.

*Step 4:* The sequence ${\overline{n}}_{h}$ is a Cauchy sequence in $L^{2}(D_T)$.

Fix a natural number *m*, define $\omega _m:=\{z\in D:d(z,\partial D)\geqslant 1/m\}$, $\omega ^T_m:=(0,T)\times \omega _m$ and write

26 $$\begin{aligned} \Vert {\overline{n}}_{h_1}-{\overline{n}}_{h_2}\Vert _{L^{2}(D_T)}&=\Vert {\overline{n}}_{h_1}-{\overline{n}}_{h_2}\Vert _{L^{2}(\omega ^T_m)}+\Vert {\overline{n}}_{h_1}-{\overline{n}}_{h_2}\Vert _{L^{2}((0,T)\times (D\setminus \omega _m))}\nonumber \\&\leqslant \Vert {\overline{n}}_{h_1}-{\overline{n}}_{h_2}\Vert _{L^{2}(\omega ^T_m)}+2\sup \limits _h\Vert n_h\Vert _{L^2((0,T)\times (D\setminus \omega _m))} \end{aligned}$$

Taking $q>1$ and using Hölder’s inequality we have

$$\begin{aligned} \Vert n_h\Vert ^2_{L^2((0,T)\times (D\setminus \omega _m))}&=\int \limits _0^{T}\int \limits _{D\setminus \omega _m} n^2_h dz dt\\&\leqslant \int \limits _0^{T}\Vert (n_h)^q \Vert _{L^2(D)} dt |D\setminus \omega _m|^{1/q^*}. \end{aligned}$$

Hence, for $q=p/2$, thanks to (25), we can conclude that $\sup \limits _h\Vert n_h\Vert _{L^2((0,T)\times (D\setminus \omega _m))}$ goes to 0 when *m* goes to infinity. Consequently, for all $\eta >0$, we can fix *m* big enough so that the second term in (26) is smaller than $\eta /2$ and then, thanks to *Step 3*, we can choose $h_1$ and $h_2$ in such a way that the first term is also smaller than $\eta /2$, which proves that $\{n_h\}_h$ is a Cauchy sequence in $L^{2}(D_T)$.

*Step 5:* Let *n* be the limit of a suitable subsequence of $n_h$ and $v:=(v^1,v^2,v^3)$ the weak limit of $v_h$ (such limit exists due to the fact that $v_h$ is bounded in $(L^2(D_T))^3$). We claim that $n\in L^2(0,T;{\mathcal {V}})$ and $v=\nabla n$.

For all $\phi (t,z)\in {\mathcal {C}}([0,T];{\mathcal {D}}(D))$, the convergence of $n_h$ to *n* implies that

27 $$\begin{aligned} \int \limits _0^T\int \limits _Dn(t,z)\partial _i\phi (t,z)dzdt=\lim \limits _{h\rightarrow 0}\int \limits _0^T\int \limits _Dn_h(t,z)\partial _i\phi (t,z)dzdt, \end{aligned}$$

where $\partial _1\phi (z)=\partial _x\phi (z)$, $\partial _2\phi (z)=\partial _y\phi (z)$ and $\partial _3\phi (z)=\partial _{\theta }\phi (z)$. Writing $\partial _i\phi (z)=\nabla \cdot (\phi (t,z) \mathbf {e_i})$, where $\{{\mathbf {e}}_i\}$ is the eucledian canonical basis, we have that

$$\begin{aligned} \int \limits _{D}n_h(t,z)\partial _i\phi (t,z)dz&=\sum \limits _{j=1}^N\nu _j(t)\int \limits _{D_j}\nabla \cdot (\phi (t,z) \mathbf {e_i})dz\\&=\sum \limits _{j=1}^N\nu _j(t)\sum \limits _{l\in N_j}\int \limits _{\Gamma _{jl}}\phi (t,z) \mathbf {e_i}\cdot \mathbf {n_{jl}}dS\\&=\sum \limits _{j=1}^N\sum \limits _{l\in N_j}\nu _j(t)\int \limits _{\Gamma _{jl}}\phi (t,z) n^i_{jl}dS. \end{aligned}$$

Therefore, we obtain

$$\begin{aligned} \int \limits _{D}n_h(t,z)\nabla \phi (t,z)dz=\sum \limits _{j=1}^N\sum \limits _{l\in N_j}\nu _j(t)\int \limits _{\Gamma _{jl}}\phi (t,z) \mathbf {n_{jl}}dS. \end{aligned}$$

Recalling that $\mathbf {z_{jl}}$ is the center of mass of $\Gamma _{jl}$, we may estimate the integral over $\Gamma _{jl}$ as

$$\begin{aligned} \int \limits _{\Gamma _{jl}}\phi (t,z) \mathbf {n_{jl}}dS=\phi (t,\mathbf {z_{jl}}) \mathbf {n_{jl}}+{\mathcal {O}}(|\Gamma _{jl}|), \end{aligned}$$

as done in (12) with the integral over $D_j$. Using the fact that $|\Gamma _{jl}|=h^2$ and the amount of cells on the mesh is ${\mathcal {O}}(h^{-3})$, we have

28 $$\begin{aligned} \int \limits _Dn_h(t,z)\nabla \phi (t,z)dz=\sum \limits _{j=1}^N\sum \limits _{l\in N_j}\nu _j(t)|\Gamma _{jl}|\phi (t,\mathbf {z_{jl}}) \mathbf {n_{jl}}+{\mathcal {O}}(h). \end{aligned}$$

Reordering the terms of this last sum, we have

29 $$\begin{aligned}&\sum \limits _{j=1}^N\sum \limits _{l\in N_j}\nu _j(t)|\Gamma _{jl}|\phi (t,\mathbf {z_{jl}}) \mathbf {n_{jl}}\nonumber \\&\quad =\frac{1}{2}\sum \limits _{j=1}^N\sum \limits _{l\in N_j}\Big (\nu _j(t)|\Gamma _{jl}|\phi (t,\mathbf {z_{jl}}) \mathbf {n_{jl}}+\nu _l(t)|\Gamma _{lj}|\phi (t,\mathbf {z_{lj}}) \mathbf {n_{lj}}\Big )\nonumber \\&\quad =-\frac{1}{2}\sum \limits _{j=1}^N\sum \limits _{l\in N_j}|\Gamma _{jl}|\mathbf {n_{jl}}\Big (\nu _l(t)-\nu _j(t)\Big )\phi (t,\mathbf {z_{jl}})\nonumber \\&\quad =-\sum \limits _{j=1}^N\sum \limits _{l\in N_j}\frac{|\Gamma _{jl}|}{|\mathbf {z_l}-\mathbf {z_j}|} (\mathbf {z_{jl}}-\mathbf {z_j})\frac{\Big (\nu _l(t)-\nu _j(t)\Big )}{s_{jl}}\phi (t,\mathbf {z_{jl}})s_{jl}\nonumber \\&\quad =-\int \limits _{D}v_h(z)\phi _h(t,z)dz, \end{aligned}$$

whereThis function strongly converges in $L^2(D_T)$ to $\phi$. Substituting (29) in (28) and then in (27), and using the weak convergence of $v_h$ to *v*, we obtain that, for all $\phi \in {\mathcal {C}}([0,T];{\mathcal {D}}(D))$

$$\begin{aligned} \int \limits _0^T\int \limits _Dn(t,z)\partial _i\phi (t,z)dzdt=-\int \limits _0^T\int \limits _Dv^i(t,z)\phi (t,z)dzdt. \end{aligned}$$

Taking $\phi (t,z)=\varphi (z)\chi (t)$, with $\varphi \in {\mathcal {D}}(D)$ and $\chi (t)\in {\mathcal {C}}([0,T])$, this last equality becomes

$$\begin{aligned} \int \limits _0^T\int \limits _{D}n(t,z)\partial _i\varphi (z)dz\chi (t) dt=-\int \limits _0^T\int \limits _{D}v^i(t,z)\varphi (z)dz\chi (t) dt, \end{aligned}$$

for all $\varphi \in {\mathcal {D}}(D)$ and $\chi (t)\in {\mathcal {C}}([0,T])$, which implies that for each $\varphi \in {\mathcal {D}}(D)$

$$\begin{aligned} \int \limits _{D}n(t,z)\partial _i\varphi (z)dz=-\int \limits _{D}v^i(t,z)\varphi (z)dz, \text{ a.e. } [0,T]. \end{aligned}$$

The separability of ${\mathcal {D}}(D)$ finally implies that

$$\begin{aligned} \int \limits _{D}n(t,z)\partial _i\varphi (z)dz=-\int \limits _{D}v^i(t,z)\varphi (z)dz,\; \forall \varphi \in {\mathcal {D}}(D), \text{ a.e. } [0,T]. \end{aligned}$$

As $v(t,z)\in L^2(D)$ for almost all $t\in [0,T]$, *n*(*t*, *z*) belongs to ${\mathcal {V}}$ for almost all $t\in [0,T]$, which proves the statements of the Proposition. $\square$

An immediate consequence of Proposition 10, together with estimate (19) is that

30 $$\begin{aligned} \Vert n\Vert ^2_{H}+a\int \limits _0^T\Vert v\Vert ^2_{H}\leqslant e^{bT}\Vert n_0\Vert ^2_{H}, \text{ for } \text{ all } T>0. \end{aligned}$$

Noticing that

$$\begin{aligned} \int \limits _0^T(\rho _h(t)-\rho (t))^2dt&=\int \limits _0^T\left( \int \limits _{D_h}n_h(t,z)dz-\int \limits _{D}n(t,z)dz\right) ^2dt\\&=\int \limits _0^T\left( \int \limits _{D_h\setminus D}n_h(t,z)dz+\int \limits _{D}(n_h(t,z)-n(t,z)dz\right) ^2dt\\&\leqslant \int \limits _0^T(|D_h\setminus D|^{1/2} \Vert n_h\Vert _{H_h}+|D|^{1/2}\Vert n_h-n\Vert _{L^2(D)})^2dt\\&\leqslant 2(|D_h\setminus D|\Vert n_h\Vert ^2_{L^2((0,T)\times D_h}+|D|\Vert n_h-n\Vert ^2_{L^2(D_T)}), \end{aligned}$$

the definition of $D_h$ and Proposition 10 implies that the sequence of functions $\rho _h(t)$ strongly converges to $\rho (t):=\int \limits _Dn(t,z)dz$ in $L^2((0,T))$.

### Existence of weak solution

This section is devoted to prove that the function *n* is a weak solution of problem (1–2).

#### Proposition 11

The function *n* satisfies

31 $$\begin{aligned} -\langle n_0,\varphi (0)\rangle =\int \limits _0^T\langle Q[n],\varphi \rangle +\langle \partial _t\varphi (t),n\rangle dt, \end{aligned}$$

for all $$\varphi \in {\mathcal {C}}^1_c([0,T),{\mathcal {V}})$$.

#### Proof

First consider $\varphi \in {\mathcal {C}}^1_c([0,T),{\mathcal {C}}^{\infty }_c({\mathbb {R}}^3))$, and for each *j*, multiply Eq. (16) by $h^3\varphi _j(t):=h^3\varphi (t,\mathbf {z_{j}})$, and add them up for all *j*, obtaining the relation

$$\begin{aligned} \sum \limits _{j=1}^Nh^3\nu '_j(t)\varphi _j(t)=A_{\varphi }(t)+D_{\varphi }(t)+R_{\varphi }(t), \end{aligned}$$

whereReordering the terms from $A_{\varphi }(t)$, we get

32 $$\begin{aligned} A_{\varphi }(t)&=\frac{1}{2}\sum \limits _{j=1}^N\sum \limits _{l\in N_j}|\Gamma _{jl}|\Big (\varphi _l(t)-\varphi _j(t)\Big )\Big (u^+_{jl}(t)\nu _j(t)+u^-_{jl}(t)\nu _l(t)\Big )\nonumber \\&=\frac{1}{2}\sum \limits _{j=1}^N\sum \limits _{l\in N_j}|\Gamma _{jl}|\Big (\varphi _l(t)-\varphi _j(t)\Big )\Big (u_{jl}(t)\nu _j(t)+u^-_{jl}(t)(\nu _l(t)-\nu _j(t))\Big )\nonumber \\&=\frac{1}{2}\sum \limits _{j=1}^N\sum \limits _{l\in N_j}|\Gamma _{jl}|\Big (\varphi _l(t)-\varphi _j(t)\Big )u_{jl}(t)\nu _j(t)+A^1_{\varphi }(t), \end{aligned}$$

where

$$\begin{aligned} A^1_{\varphi }(t)=\frac{1}{2}\sum \limits _{j=1}^N\sum \limits _{l\in N_j} u^-_{jl}(t)|\Gamma _{jl}|\Big (\varphi _l(t)-\varphi _j(t)\Big )\Big (\nu _l(t)-\nu _j(t)\Big ). \end{aligned}$$

Thanks to the boundedness of $u_{jl}$ and the regularity of $\varphi$, this term satisfies

33 $$\begin{aligned} |A^1_{\varphi }(t)|&\leqslant \frac{{\overline{V}}}{2}\Vert \nabla \varphi \Vert _{L^{\infty }({\mathbb {R}}^3)}h\sum \limits _{j=1}^N\sum \limits _{l\in N_j} |\Gamma _{jl}||\nu _l(t)-\nu _j(t)|\nonumber \\&=\frac{h}{6}{\overline{V}}\Vert \nabla \varphi \Vert _{L^{\infty }({\mathbb {R}}^3)}\Vert v_h\Vert _{L^1(D_h)}\nonumber \\&\leqslant \frac{h}{6}|D_h|^{1/2} {\overline{V}}\Vert \nabla \varphi \Vert _{L^{\infty }({\mathbb {R}}^3)}\Vert v_h\Vert _{L^2(D_h)}. \end{aligned}$$

Recalling the definition of $u_{jl}$ and the property (8), we get from (32)

$$\begin{aligned} A_{\varphi }(t)&=\frac{1}{2}\sum \limits _{j=1}^N\sum \limits _{l\in N_j}|\Gamma _{jl}|\frac{\Big (\varphi _l(t)-\varphi _j(t)\Big )}{|\mathbf {z_l}-\mathbf {z_j}|}V_{jl}(t)\cdot (\mathbf {z_l}-\mathbf {z_j})\nu _j(t)+A^1_{\varphi }(t),\\&=\sum \limits _{j=1}^N\sum \limits _{l\in N_j}|\Gamma _{jl}|\frac{\Big (\varphi _l(t)-\varphi _j(t)\Big )}{|\mathbf {z_l}-\mathbf {z_j}|}V_{jl}(t)\cdot (\mathbf {z_{jl}}-\mathbf {z_j})\nu _j(t)+A^1_{\varphi }(t), \end{aligned}$$

Defining $V_j(t):=V(t,\mathbf {z_j})$ allows us to write

$$\begin{aligned} A_{\varphi }(t)=\sum \limits _{j=1}^N\sum \limits _{l\in N_j}|\Gamma _{jl}|\frac{\Big (\varphi _l(t)-\varphi _j(t)\Big )}{|\mathbf {z_l}-\mathbf {z_j}|}V_{j}(t)\cdot (\mathbf {z_{jl}}-\mathbf {z_j})\nu _j(t)+A^1_{\varphi }+A^2_{\varphi }(t),\\ \end{aligned}$$

with

$$\begin{aligned} A^2_{\varphi }(t)=\sum \limits _{j=1}^N\sum \limits _{l\in N_j}|\Gamma _{jl}|\frac{\Big (\varphi _l(t)-\varphi _j(t)\Big )}{|\mathbf {z_l}-\mathbf {z_j}|}(V_{jl}(t)-V_{j}(t))\cdot (\mathbf {z_{jl}}-\mathbf {z_j})\nu _j(t). \end{aligned}$$

Thanks to the regularity of $\varphi$ and *V*, we have

34 $$\begin{aligned} |A^2_{\varphi }(t)|&\leqslant \frac{h}{4}\Vert \nabla \varphi \Vert _{L^{\infty }({\mathbb {R}}^3)}\Vert \nabla V\Vert _{L^{\infty }({\mathbb {R}}^3)}\sum \limits _{j=1}^N\sum \limits _{l\in N_j}h^3\nu _j(t)\nonumber \\&\leqslant \frac{h}{4}|D_h|^{1/2}\Vert \nabla \varphi \Vert _{L^{\infty }({\mathbb {R}}^3)}\Vert \nabla V\Vert _{L^{\infty }({\mathbb {R}}^3)}\Vert n_h\Vert _{L^2(D_h)}. \end{aligned}$$

Finally, we write

$$\begin{aligned} A_{\varphi }(t)=&\sum \limits _{j=1}^N\sum \limits _{l\in N_j}|\Gamma _{jl}|\Big (\nabla \varphi (\mathbf {z_j})\cdot \mathbf {n_{jl}}\Big )V_{j}(t)\cdot (\mathbf {z_{jl}}-\mathbf {z_j})\nu _j(t)\\&+A^1_{\varphi }(t)+A^2_{\varphi }(t)+A^3_{\varphi }(t)\\ :=&A^0_{\varphi }(t)+A^1_{\varphi }(t)+A^2_{\varphi }(t)+A^3_{\varphi }(t), \end{aligned}$$

where

$$\begin{aligned} A^3_{\varphi }(t)=\sum \limits _{j=1}^N\sum \limits _{l\in N_j}|\Gamma _{jl}|\left( \frac{\Big (\varphi _l(t)-\varphi _j(t)\Big )}{|\mathbf {z_l}-\mathbf {z_j}|}-\nabla \varphi (\mathbf {z_j})\cdot \mathbf {n_{jl}}\right) V_{j}(t)\cdot (\mathbf {z_{jl}}-\mathbf {z_j})\nu _j(t). \end{aligned}$$

Again, the regularity of $\varphi$ and the boundedness of *V* imply

$$\begin{aligned} |A^3_{\varphi }|\leqslant&\frac{h}{4}{\overline{V}}|D_h|^{1/2}\Vert \nabla ^2\varphi \Vert _{L^{\infty }({\mathbb {R}}^3)}\Vert n_h\Vert _{L^2(D_h)}. \end{aligned}$$

And this together with (33), (34) and estimate (19) ensures that there exists a constant $C_A$ independent of *h* such that

$$\begin{aligned} \int \limits _0^T|A^1_{\varphi }(t)+A^2_{\varphi }(t)+A^3_{\varphi }(t)|dt\leqslant C_Ah. \end{aligned}$$

On the other hand, we can rewrite the remaining term as

$$\begin{aligned} A^0_{\varphi }(t)&=\sum \limits _{j=1}^N\sum \limits _{l\in N_j}|\Gamma _{jl}|\Big (\nabla \varphi (\mathbf {z_j})\cdot \mathbf {n_{jl}}\Big )V_{j}(t)\cdot (\mathbf {z_{jl}}-\mathbf {z_j})\nu _j(t)\\&=\sum \limits _{j=1}^N\nu _j(t)V_{j}(t) \cdot \sum \limits _{l\in N_j}|\Gamma _{jl}|\Big (\nabla \varphi (\mathbf {z_j})\cdot \mathbf {n_{jl}}\Big )(\mathbf {z_{jl}}-\mathbf {z_j}). \end{aligned}$$

In Eymard et al. (2006), it was proven that

$$\begin{aligned} \sum \limits _{l\in N_j}|\Gamma _{jl}|\Big (\nabla \varphi (\mathbf {z_j})\cdot \mathbf {n_{jl}}\Big )(\mathbf {z_{jl}}-\mathbf {z_j})=h^3\nabla \varphi (\mathbf {z_j}), \end{aligned}$$

consequently

$$\begin{aligned} A^0_{\varphi }(t)=\sum \limits _{j=1}^N\nu _j(t)V_{j}(t) \cdot \nabla \varphi (\mathbf {z_j})h^3=\int \limits _{D}n_h(t)\Big (V\cdot \nabla \varphi \Big )_h(t),\nonumber \end{aligned}$$

whereMoreover, the sequence $\Big (V\cdot \nabla \varphi \Big )_h(t)$ strongly converges to $V\cdot \nabla \varphi$ in $L^2(D_T)$, which implies that

35 $$\begin{aligned} \int \limits _0^TA_{\varphi }(t)dt\longrightarrow \int \limits _0^T\int \limits _D nV\cdot \nabla \varphi dzdt, \text{ for } \text{ all } \varphi \in {\mathcal {C}}^1_c([0,T),{\mathcal {C}}^{\infty }_c({\mathbb {R}}^3)). \end{aligned}$$

Reordering as well the terms from $D_{\varphi }$, we get

$$\begin{aligned} D_{\varphi }(t)&=-\frac{1}{2}\sum \limits _{j=1}^N\sum \limits _{l\in N_j}\frac{A_{jl}|\Gamma _{jl}|}{|\mathbf {z_l}-\mathbf {z_j}|}\Big (\nu _l(t)-\nu _j(t) \Big )\Big (\varphi _l(t)-\varphi _j(t)\Big )\\&=-\frac{1}{2}\sum \limits _{j=1}^N\sum \limits _{l\in N_j}\frac{A_{jl}|\Gamma _{jl}|}{|\mathbf {z_l}-\mathbf {z_j}|}\Big (\nu _l(t)-\nu _j(t) \Big )\Big (\nabla \varphi (\mathbf {z_{jl}})\cdot (\mathbf {z_l}-\mathbf {z_j})\Big )+D^1_{\varphi }(t)\\&=-\sum \limits _{j=1}^N\sum \limits _{l\in N_j}\frac{A_{jl}|\Gamma _{jl}|}{|\mathbf {z_l}-\mathbf {z_j}|}\Big (\nu _l(t)-\nu _j(t) \Big )\Big (\nabla \varphi (\mathbf {z_{jl}})\cdot (\mathbf {z_{jl}}-\mathbf {z_j})\Big )+D^1_{\varphi }(t) \end{aligned}$$

where

$$\begin{aligned} D^1_{\varphi }(t)=-\frac{1}{2}\sum \limits _{j=1}^N\sum \limits _{l\in N_j}\frac{A_{jl}|\Gamma _{jl}|}{|\mathbf {z_l}-\mathbf {z_j}|}\Big (\nu _l(t)-\nu _j(t) \Big )\Big ( \varphi _l(t)-\varphi _j(t)-\nabla \varphi (\mathbf {z_{jl}})\cdot (\mathbf {z_l}-\mathbf {z_j})\Big ). \end{aligned}$$

Thanks to the boundedness of the coefficients $A_{jl}$ and the regularity of $\varphi$ we get

36 $$\begin{aligned} |D^1_{\varphi }|&\leqslant h|D_h|^{1/2}\frac{{\overline{\alpha }}}{4}\Vert \nabla ^2\varphi \Vert _{L^{\infty }({\mathbb {R}}^3)}\Vert v_h\Vert _{L^2(D_h)}. \end{aligned}$$

Recalling the definition of $A_{jl}$, the fact that $A(\theta )$ is a diagonal matrix and the normal vectors to the boundary of $D_j$ are elements of the canonical euclidean basis or their opposites, we have that, for all *j* and *l*

$$\begin{aligned} A_{jl}\Big (\nabla \varphi (\mathbf {z_{jl}})\cdot (\mathbf {z_{jl}}-\mathbf {z_j})\Big )=\Big (A(\theta _{jl})\nabla \varphi (\mathbf {z_{jl}})\Big )\cdot (\mathbf {z_{jl}}-\mathbf {z_j}). \end{aligned}$$

Consequently, we infer that

$$\begin{aligned} D_{\varphi }(t)=-\int \limits _{D} v_h\cdot \Big (A(\theta )\nabla \varphi (z)\Big )_h dz+D^1_{\varphi }(t), \end{aligned}$$

wherestrongly converges to $A(\theta )\nabla \varphi (z)$ in $\Big (L^2(D_T)\Big )^3$. The regularity of $\varphi$, the boundedness of $v_h$ in $L^2(D_T)$ and (36) guarantee the existence of $C_D$ such that

$$\begin{aligned} \int \limits _0^T|D^1_{\varphi }(t)|dt\leqslant C_Dh, \end{aligned}$$

so that, consequently,

37 $$\begin{aligned} \int \limits _{0}^TD(t)dt\longrightarrow \int \limits _0^T\int \limits _DA(\theta )\nabla n\nabla \varphi dzdt, \text{ for } \text{ all } \varphi \in {\mathcal {C}}^1_c([0,T),{\mathcal {C}}^{\infty }_c({\mathbb {R}}^3)). \end{aligned}$$

The sequence of functions

$$\begin{aligned} R_h(t,z)=\sum \limits _{j=1}^N\Big (r_j-d_j\rho _h(t)\Big )\mathbb {1}_{D_j}(z), \end{aligned}$$

belongs to $L^{\infty }(D_T)$ and strongly converges in $L^2(D_T)$ to $r(z)-d(z)\rho (t)$, which implies that

38 $$\begin{aligned} \int _0^TR_{\varphi }(t)dt\longrightarrow \int \limits _0^T\int \limits _{D}\Big (r(z)-d(z)\rho (t)\Big )n\varphi dt, \text{ for } \text{ all } \varphi \in {\mathcal {C}}^1_c([0,T),{\mathcal {C}}^{\infty }_c({\mathbb {R}}^3)).\nonumber \\ \end{aligned}$$

Finally, we conclude that

$$\begin{aligned} \int \limits _{0}^T\sum \limits _{j=1}^Nh^3\nu '_j(t)\varphi _j(t)dt&=\sum \limits _{j=1}^Nh^3\int \limits _{0}^T\nu '_j(t)\varphi _j(t)dt\\&=-\sum \limits _{j=1}^Nh^3\left( \nu _j(0)\varphi _j(0)+\int \limits _{0}^T\nu _j(t)\varphi '_j(t)dt\right) , \end{aligned}$$

which converges to

$$\begin{aligned} -\int \limits _Dn_0\varphi (0)dz-\int \limits _{0}^T\int \limits _{D}n(t,z)\partial _t\varphi (t,z)dzdt. \end{aligned}$$

Putting this result together with (35), (37) and (38), we get that, for all $\varphi \in {\mathcal {C}}^1_c([0,T),{\mathcal {C}}^{\infty }_c({\mathbb {R}}^3))$

39 $$\begin{aligned} -\langle n_0,\varphi (0)\rangle =\int \limits _0^T\langle Q[n],\varphi \rangle +\langle \partial _t\varphi (t),n\rangle dt. \end{aligned}$$

As ${\mathcal {C}}^1_c([0,T),{\mathcal {C}}^{\infty }_c({\mathbb {R}}^3))$ is dense in ${\mathcal {C}}^1_c([0,T),H^1(\omega ))$ for all compacts $\omega \subset D$, and the functions *n* and $\nabla n$ belong to $L^2(D_T)$, (39) also holds for all $\varphi \in {\mathcal {C}}^1_c([0,T),{\mathcal {V}})$. $\square$

#### Proposition 12

The function *n* belongs to $H^1((0,T);{\mathcal {V}}')$.

#### Proof

Taking $\varphi :=\chi (t)\psi (z)$ with $\chi \in {\mathcal {D}}((0,T))$ and $\psi \in {\mathcal {V}}$ in Eq. (31), we get

$$\begin{aligned} \Big \langle \int \limits _0^T n \chi 'dt,\psi \Big \rangle =&\int \limits _0^T\langle \psi \chi ',n\rangle dt={\int \limits _0^T\langle \partial _t\varphi ,n\rangle dt}\\ =&-\int \limits _0^T\langle Q[n],\varphi \rangle dt=-\langle \int \limits _0^T Q[ n] \chi dt,\psi \rangle . \end{aligned}$$

As this holds true for all $\psi \in {\mathcal {V}}$, this equation is equivalent with

$$\begin{aligned} \int \limits _0^T n \chi 'dt=-\int \limits _0^T Q[ n]\chi dt \text{ in } {\mathcal {V}}' \text{ for } \text{ any } \chi \in {\mathcal {D}}((0,T)). \end{aligned}$$

or in other words

$$\begin{aligned} \partial _t n=Q [n] \text{ in } \text{ the } \text{ sense } \text{ of } \text{ distributions } \text{ in } {\mathcal {V}}'. \end{aligned}$$

The estimate (30) implies that $Q[ n]\in L^2((0,T);{\mathcal {V}}')$, consequently, *n* belongs to $H^1((0,T);{\mathcal {V}}')$. $\square$

#### Proposition 13

The function *n* belongs to ${\mathcal {C}}([0,T],L^2(D))$.

#### Proof

From Proposition 8 we have that $n\in L^2((0,T),{\mathcal {V}})$. Define ${\overline{n}}(t,z)=n(t,z)\mathbb {1}_{[0,T]}(t)$ and the approximation to the identity sequence

$$\begin{aligned} \Phi _{\varepsilon }(t):=\varepsilon ^{-1}\Phi (\varepsilon ^{-1}t), \end{aligned}$$

where $\Phi (t)$ is a mollifier with compact support included in $(-1,-1/2)$. The sequence $n_{\varepsilon }(t):=n*_t\Phi _{\varepsilon }$ belongs to ${\mathcal {C}}^1({\mathbb {R}},{\mathcal {V}})$, $n_{\varepsilon }\rightarrow n$ a.e. on [0, *T*] and in $L^2((0,T),{\mathcal {V}})$. For a fixed $\tau \in (0,T)$ and for any $t\in (0,\tau )$ and any $0<\varepsilon <T-\tau$, we have $s\rightarrow \Phi _{\varepsilon }(t-s)\in {\mathcal {D}}(0,T)$, since $\text{ supp } \Phi _{\varepsilon }(t-\cdot )\subset [t+\varepsilon /2,t+\varepsilon ]\subset [\varepsilon /2,\tau +\varepsilon ]$. Therefore, we get

$$\begin{aligned} n'_{\varepsilon }&=\int \limits _{{\mathbb {R}}}\partial _{t}\Phi _{\varepsilon }(t-s){\overline{n}}(s)ds\\&=-\int \limits _{0}^T\partial _s\Phi _{\varepsilon }(t-s)n(s)ds =\int \limits _{0}^T\Phi _{\varepsilon }(t-s)n'(s)ds=\Phi _{\varepsilon }*_t\overline{n'}. \end{aligned}$$

As a consequence $n'_{\varepsilon }\rightarrow n'$ a.e. and in $L^2((0,\tau ),{\mathcal {V}}')$. Now fix $\tau \in (0,T)$ and $\varepsilon ,\varepsilon '\in (0,T-\tau )$, and compute

$$\begin{aligned} \frac{d}{dt}\Vert n_{\varepsilon }(t)-n_{\varepsilon '}(t)\Vert ^2_H=2\langle n'_{\varepsilon }-n'_{\varepsilon '},n_{\varepsilon }-n_{\varepsilon '}\rangle _{{\mathcal {V}}'\times {\mathcal {V}}}, \end{aligned}$$

so that for any $t_1,t_2\in [0,\tau ]$

$$\begin{aligned} \Vert n_{\varepsilon }(t_2)-n_{\varepsilon '}(t_1)\Vert ^2_H=\Vert n_{\varepsilon }(t_1)-n_{\varepsilon '}(t_1)\Vert ^2_H+2\int \limits _{t_1}^{t_2}\langle n'_{\varepsilon }-n'_{\varepsilon '},n_{\varepsilon }-n_{\varepsilon '}\rangle dt . \end{aligned}$$

Since $n_{\varepsilon }\rightarrow n$ a.e. in $[0,\tau ]$ in *V*, fix $t_1$ such that $n_{\varepsilon }(t_1)\rightarrow n(t_1)$ in *V*, so as a consequence of $n_{\varepsilon }\rightarrow n$ in $L^2((0,T),{\mathcal {V}})$ and $n'_{\varepsilon }\rightarrow n'$ in $L^2((0,\tau ),{\mathcal {V}}')$ we have

$$\begin{aligned} \limsup \limits _{\varepsilon ,\varepsilon '\rightarrow 0}\sup \limits _{[0,\tau ]}\Vert n_{\varepsilon }-n_{\varepsilon '}\Vert ^2_H\leqslant \lim \limits _{\varepsilon ,\varepsilon '\rightarrow 0}\int \limits _0^{\tau }\Vert n'_{\varepsilon }-n'_{\varepsilon '}\Vert _{{\mathcal {V}}'} \Vert n_{\varepsilon }-n_{\varepsilon '}\Vert _{{\mathcal {V}}}d dt=0. \end{aligned}$$

So that $n_{\varepsilon }$ is a Cauchy sequence in ${\mathcal {C}}([0,\tau ],L^2(D))$, and then $n_{\varepsilon }$ converges in ${\mathcal {C}}([0,\tau ],L^2(D))$ to a limit ${\tilde{n}}\in {\mathcal {C}}([0,\tau ],L^2(D))$. That proves $n={\tilde{n}}$ a.e. and $n\in {\mathcal {C}}([0,\tau ],L^2(D))$. By taking $\Phi (-t)$ as the mollifier function in the previous proof, and choosing $\tau \in (0,T)$ and $\varepsilon \in (0,\tau )$ can be proven that $n\in {\mathcal {C}}([\tau ,T],L^2(D))$, and consequently $n\in {\mathcal {C}}([0,T],L^2(D))$. $\square$

#### Proposition 14

The function *n* is a weak solution of problem (1–2).

#### Proof

Assume first $\varphi \in {\mathcal {C}}_c([0,T),H)\cap L^2((0,T),{\mathcal {V}})\cap H^1([0,T],{\mathcal {V}}')$. We define $\varphi _{\varepsilon }(t)=\varphi *_t \Phi _{\varepsilon }$ for a mollifier $\Phi _{\varepsilon }$ with compact support included in $(0,\infty )$ so that $\varphi _{\varepsilon }\in {\mathcal {C}}^1_c([0,T);{\mathcal {V}})$ for any $\varepsilon >0$ small enough and

$$\begin{aligned} \varphi _{\varepsilon } \rightarrow \varphi \text{ in } {\mathcal {C}}([0,T],H)\cap L^2((0,T),{\mathcal {V}})\cap H^1([0,T],{\mathcal {V}}'). \end{aligned}$$

Writing the Eq. (31) for $\varphi _{\varepsilon }$ and passing to the limit $\varepsilon \rightarrow 0$ we get that (31) also holds true for $\varphi$.

Assume now $\varphi \in X_T=C([0,T],H)\cap L^2((0,T),{\mathcal {V}})\cap H^1([0,T],{\mathcal {V}}')$. Fix $\chi \in {\mathcal {C}}^1({\mathbb {R}})$, such that $\text{ supp }\, \chi \subset (-\infty ,0)$, $\chi '\leqslant 0$, $\chi '\in {\mathcal {C}}_c((-1,0))$, and such that the integral of $\chi '$ is $-1$. For example, fix $\delta <1/2$ and define

$$\begin{aligned} \chi (s)=\left\{ \begin{array}{lcr} 1 &{} \text{ if } &{} s\leqslant -1+\delta \\ &{}&{}\\ \frac{1}{2}(1+\cos (\frac{\pi (t+1-\delta )}{1-2\delta }))&{} \text{ if } &{} -1+\delta \leqslant s\leqslant -\delta \\ &{}&{}\\ 0 &{} \text{ if } &{} s\geqslant -\delta \end{array} \right. \end{aligned}$$

Now define $\chi ^t_{\varepsilon }=\chi (\frac{s-t}{\varepsilon })$, so that $\varphi _{\varepsilon }:=\varphi \chi ^t_{\varepsilon }\in {\mathcal {C}}_c([0,T);H)$ and $\chi ^t_{\varepsilon }\rightarrow {\mathbf {1}}_{[0,t]}$, $(\chi ^t_{\varepsilon })'\rightarrow -\delta _t$ as $\varepsilon \rightarrow 0$. Equation (31) for the function $\varphi _{\varepsilon }$ writes

$$\begin{aligned} -(n_0,\varphi (0))-\int \limits _{0}^T(n,\varphi (s))(\chi ^t_{\varepsilon })'ds=\int \limits _0^T\chi ^t_{\varepsilon }\Big (\langle Q [n](s),\varphi (s)\rangle +\langle \varphi '(s),n\rangle \Big )ds.\nonumber \end{aligned}$$

Passing to the limit when $\varepsilon$ goes to 0 we obtain that *n* is a solution for the variational formulation. $\square$

### A discrete implicit scheme

Once we have established the proof of existence of a solution for problem (16–17), together with the obtention of a semi-discrete scheme in order to approximate such solution, we proceed to derive an implicit discrete scheme starting from problem (16–17), and to prove its convergence.

Consider a natural number *K* and define $\Delta t=\frac{T}{K}$ and $t_k=k\Delta t$, $\nu ^k_j:=\nu _j(t_k)$, $k=1,\ldots ,K$. Using a forward difference approximation for the time derivative in (16) we get the implicit scheme

40 $$\begin{aligned} \frac{\nu ^{k+1}_j-\nu ^k_j}{\Delta t}=M^{k+1}_{j}\nu ^{k+1}_j+\sum \limits _{l\in N_j}B^{k+1}_{jl}\nu ^{k+1}_l, \end{aligned}$$

where

$$\begin{aligned} M^{k+1}_j&:=M_{j} (t_{k+1})=-\frac{|\Gamma _{jl}|}{h^3}\sum \limits _{l\in N_j}\Big (u^+_{jl}(t_{k+1})+\frac{A_{jl}}{|\mathbf {z_l}-\mathbf {z_j}|}\Big )+\Big (r_j-d_j\sum \limits _{l=1}^Nh^3\nu ^{k+1}_l\Big ),\\ B^{k+1}_{jl}&:=B_{jl}(t_{k+1})=\frac{|\Gamma _{jl}|}{h^3}\Big (-u^-_{jl}(t_{k+1})+\frac{A_{jl}}{|\mathbf {z_l}-\mathbf {z_j}|}\Big ). \end{aligned}$$

#### Theorem 15

Let $\nu ^0_j$ be non-negative initial data with mass $\rho _0=\sum \limits _{j=1}^Nh^3\nu ^0_j$ and assume that

41 $$\begin{aligned} \Delta t<\frac{1}{\left( \sqrt{r^++d^+{\overline{\rho }}}+\sqrt{d^+{\overline{\rho }}}\right) ^2}, \end{aligned}$$

then there exists a unique non-negative solution $\nu ^k_j$, $k=1,\ldots ,N$ to scheme (40). Furthermore, for each *h*, the sequence of piecewise constant functions

$$\begin{aligned} \nu ^j_{\Delta t}(t)=\sum \limits _{k=0}^{K}\nu ^k_{j}\mathbb {1}_{(t_k,t_{k+1})}, \end{aligned}$$

strongly converges to the solution of (16–17) in $(L^2((0,T)))^N$ when $\Delta t$ goes to 0.

#### Proof

For all $\Delta t$ satisfying (41), there exists $\lambda <1$ such that

42 $$\begin{aligned} \Delta t(r^++d^+{\overline{\rho }}_{\lambda })<\lambda , \end{aligned}$$

where ${\overline{\rho }}_{\lambda }=\frac{{\overline{\rho }}}{1-\lambda }$. Consider the set

$$\begin{aligned} {\mathcal {X}}=\{\eta \in {\mathbb {R}}^M:\eta _j\geqslant 0\,\forall j,\;\Vert \eta \Vert _1=\sum \limits _jh^3\eta _j\leqslant {\overline{\rho }}_{\lambda }\}, \end{aligned}$$

and assume $\nu ^k\in {\mathbb {R}}^M$ to be the solution of (40) for a previous iteration, having all non-negative components and satisfying $\sum \limits _jh^3\nu ^n_j\leqslant {\overline{\rho }}$. Define the operator $\nu =F(\eta ):{\mathcal {X}}\rightarrow {\mathbb {R}}^M$ as the solution of the linear system

43 $$\begin{aligned} {P}(\eta )\nu =\nu ^k, \end{aligned}$$

where the components of matrix $M(\eta )$ are defined as

$$\begin{aligned} P_{jl}(\eta )=\left\{ \begin{array}{lll} -\Delta t B^{n+1}_{jl},&{} \text{ if } l\in N_j,\\ &{}\\ 1+\Delta t\Big (\frac{|\Gamma _{jl}|}{h^3}\sum \limits _{l\in N_j}\Big (u^+_{jl}(t_{n+1})+\frac{A_{jl}}{|\mathbf {z_l}-\mathbf {z_j}|}\Big )-\Big (r_j-d_j\Vert \eta \Vert _1\Big )\Big )&{} \text{ if } j=l. \end{array} \right. \end{aligned}$$

From the definition of ${\mathcal {X}}$ and the choice of $\Delta t$ we have that $P_{jl}(\eta )$ is positive for all $\eta$ if $j=l$, and non-positive if $j\ne l$. Furthermore $P(\eta )$ is a column-dominant matrix, so that we may conclude that $P(\eta )$ is an *M*-matrix, which implies that its inverse exists and has only non-negative entries. As $\nu ^k$ has all non-negative components, then $\nu =F(\eta )$ also has non-negative components for all $\eta \in {\mathcal {X}}$. Multiplying system (43) by $h^3$ and adding up all equations, thanks to (42) we obtain

$$\begin{aligned} \sum \limits _{j=1}^Nh^3\nu _j=&\sum \limits _{j=1}^Nh^3\nu ^k_j+\Delta t\sum \limits _{j=1}^Nh^3\Big (r_j-d_j\Vert \eta \Vert _1\Big )\nu _j\\&\leqslant {\overline{\rho }}+\Delta t(r^++d^+{\overline{\rho }}_{\lambda })\sum \limits _{j=1}^Nh^3\nu _j\\&< {\overline{\rho }}+\lambda \sum \limits _{j=1}^Nh^3\nu _j. \end{aligned}$$

This implies that $\sum \limits _{j=1}^Nh^3\nu _j<{\overline{\rho }}_{\lambda }$ and consequently, $F(\eta )$ is a continuous application going from ${\mathcal {X}}$ to itself, and thanks to Brouwer’s fixed point theorem, $F(\eta )$ has at least a fixed point on ${\mathcal {X}}$. Furthermore, a fixed point of $F(\eta )$ will satisfy

$$\begin{aligned} \sum \limits _{j=1}^Nh^3\nu _j=\sum \limits _{j=1}^Nh^3\nu ^k_j+\Delta t\sum \limits _{j=1}^Nh^3\Big (r_j-d_j\Vert \nu \Vert _1\Big )\nu _j \end{aligned}$$

which in turn implies $\Vert \nu \Vert _1\leqslant {\overline{\rho }}$. As a consequence, the implicit Euler scheme satisfies

44 $$\begin{aligned} \Vert \nu ^k\Vert _1\leqslant {\overline{\rho }} \qquad \text{ for } \text{ all } k. \end{aligned}$$

For the uniqueness, assume there exists two different solutions $\nu$ and $\mu$ to scheme (40). Let us denote the sign of $\beta \in {\mathbb {R}}$ as $sign(\beta )$. Taking the difference for each equation, multiplying by $h^3 sign(\nu _j-\mu _j)$, adding up all the equations and recalling that

$$\begin{aligned} \sum \limits _{l\in N_j}B^{n+1}_{jl}=\frac{|\Gamma _{jl}|}{h^3}\sum \limits _{l\in N_j}\Big (u^+_{jl}(t_{n+1})+\frac{A_{jl}}{|\mathbf {z_l}-\mathbf {z_j}|}\Big ), \end{aligned}$$

we obtain that

$$\begin{aligned} \sum \limits _jh^3|\nu _j-\mu _j|=&\Delta t\sum \limits _jh^3\Big ((r_j-d_j\Vert \nu \Vert _1)|\nu _j-\mu _j|\\&+d_j sign(\nu _j-\mu _j)(\Vert \nu \Vert _1-\Vert \mu \Vert _1)\mu _j\Big )\\&\leqslant \Delta t(r^++d^+{\overline{\rho }})\sum \limits _jh^3|\nu _j-\mu _j|. \end{aligned}$$

The condition over $\Delta t$ then implies that $\Vert \nu -\mu \Vert _1=0$ and consequently $\nu =\mu$.

To prove the convergence of $\nu ^j_{\Delta t}$ to $\nu _j$, we define the sequences of continuous functions

$$\begin{aligned} \mu ^j_{\Delta t}(t)=\sum \limits _{k=1}^{K}\frac{(t-t_k)\nu ^{k+1}_{j}+(t_{k+1}-t)\nu ^k_j}{\Delta t}\mathbb {1}_{(t_k,t_{k+1})}. \end{aligned}$$

These sequences are in ${\mathcal {C}}([0,T])$ and are uniformly bounded because for all values of *j* and *k*, $\nu ^k_j\leqslant \frac{{\overline{\rho }}}{h^3}$ due to (44). Furthermore, thanks to (40)

$$\begin{aligned} |\mu ^j_{\Delta t}-\nu ^j_{\Delta t}|\leqslant \max \limits _{k}|\nu ^{k+1}_j-\nu ^k_j|=\Delta t\max \limits _{k}|M^{k+1}_{j}\nu ^{k+1}_j+\sum \limits _{l\in N_j}B^{k+1}_{jl}\nu ^{k+1}_l|\leqslant C\Delta t , \end{aligned}$$

where *C* is independent of $\Delta t$. Consequently, for each *j*, when $\Delta t$ goes to 0 both sequences $\nu ^j_{\Delta t}$ and $\mu ^j_{\Delta t}$ strongly converge in $L^2((0,T))$ to a certain continuous functions $\nu ^*_j(t)$.

We consider now a function $\varphi \in {\mathcal {C}}^1_0((0,T))$ and we define $\varphi _k:=\varphi (t_k)$. For all *k*, multiply (40) by $\Delta t\varphi _k$ and add over *k* in order to obtain

$$\begin{aligned} \sum \limits _{k=1}^{K}(\nu ^{k}_j-\nu ^{k-1}_j)\varphi _k=\sum \limits _{k=1}^{K}\Delta t\Big (M^{k}_{j}\nu ^{k}_j+\sum \limits _{l\in N_j}B^k_{jl}\nu ^{k}_l\Big )\varphi _k, \end{aligned}$$

or, after reordering the sum on the left side

45 $$\begin{aligned} \sum \limits _{k=1}^{K}\Delta t\nu ^{k}_j\frac{\varphi _k-\varphi _{k-1}}{\Delta t}=\sum \limits _{k=1}^{K}\Delta t\Big (M^{k}_{j}\nu ^{k}_j+\sum \limits _{l\in N_j}B^k_{jl}\nu ^{k}_l\Big )\varphi _k. \end{aligned}$$

For all $\varphi$ in ${\mathcal {C}}^1_0((0,T))$, the sequence

$$\begin{aligned} \sum \limits _{k=1}^{K}\frac{\varphi _k-\varphi _{k-1}}{\Delta t}\mathbb {1}_{(t_k,t_{k+1})}, \end{aligned}$$

strongly converges in $L^2((0,T))$ to $\varphi '$. The boundedness of the coefficients $M^{k}_{j}$ and $B^k_{jl}$ together with strong convergence of $\nu ^j_{\Delta t}$ imply that

$$\begin{aligned} \sum \limits _{k=1}^{K}\Big (M^{k}_{j}\nu ^{k}_j+\sum \limits _{l\in N_j}B^k_{jl}\nu ^{k}_l\Big )\mathbb {1}_{(t_k,t_{k+1})}, \end{aligned}$$

strongly converges to $M_{j}(t,\rho _h(t))\nu ^{*}_j+\sum \limits _{l\in N_j}B_{jl}(t)\nu ^{*}_l$, so, taking the limit in (45), we get

$$\begin{aligned} \int \limits _0^T\nu ^{*}_j\varphi '(t)dt=\int \limits _0^T \Big (M_{j}(t,\nu ^*_j)\nu ^{*}_j+\sum \limits _{l\in N_j}B_{jl}(t)\nu ^{*}_l\Big )\varphi (t) dt, \end{aligned}$$

which implies that $\nu ^*_j$ is in ${\mathcal {C}}^1$ and is a solution for (16). Furthermore, as $\nu ^*_j$ is the point-wise limit of $\mu ^j_{\Delta t}$, it also satisfies the initial conditions (17). $\square$

## Simulations

The first part of this section is devoted to the numerical analysis of the approximation error. For certain values of the coefficients of problem (1–3), it is possible to obtain an analytical solution, which we will use in order to compare with our numerical approximation.

Assume that $r(x,y,\theta )$ and $d(x,y,\theta )$ are constants, that $V(t,x,y,\theta )$ is independent of *t* and that exists $W(x,y,\theta )$ such that

$$\begin{aligned} V(z)=A(\theta )\nabla W(z). \end{aligned}$$

Assume as well that

$$\begin{aligned} n_0(z)=C\frac{e^{W(z)}}{\int \limits _De^{W(z)}dz}. \end{aligned}$$

Then, the solution for problem (1–3) is

$$\begin{aligned} n(t,z)=\frac{e^{W(z)}}{\int \limits _De^{W(z)}dz}\frac{re^{rt}}{d(K+e^{rt})}, \end{aligned}$$

where

$$\begin{aligned} K=\frac{r-Cd}{Cd}. \end{aligned}$$

The existence of an analytic solution allows us to compare its values with those obtained from solving (16) for different values of *h*, and this way, numerically establish the error order of the method. Choosing

$$\begin{aligned} D:= & {} \{(x,y,\theta )\in [0,1]^3:x^2+y^2\leqslant 1\}, \\ V(t,z)= & {} \begin{pmatrix} -(\theta +1)x\\ -(\theta +1)y\\ 1 \end{pmatrix},\; A(\theta )=\begin{pmatrix} \theta +1&{}0&{}0\\ 0&{}\theta +1&{}0\\ 0&{}0&{}1 \end{pmatrix}, \end{aligned}$$

$r=d=1$ and $n_0=\Big (\pi (1-e^{-1/2})(e-1)\Big )^{-1}e^{-\frac{x^2}{2}-\frac{y^2}{2}+\theta }$, the exact solution for (1–3) is

$$\begin{aligned} n(t,z)=\Big (\frac{\pi }{2}(1-e^{-1/2})(e-1)\Big )^{-1}e^{-\frac{x^2}{2}-\frac{y^2}{2}+\theta }\frac{e^{t}}{1+e^{t}}. \end{aligned}$$

**Fig. 1:**
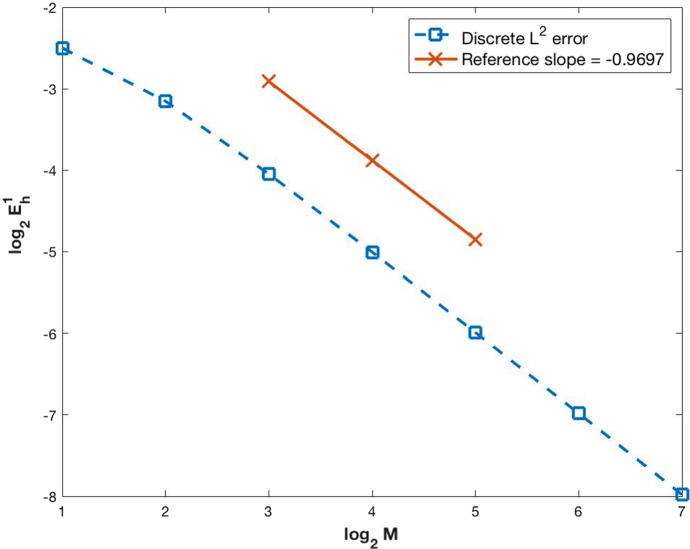
The discrete $L^2(D_T)$ error for the semi-discrete scheme, for $T=10$ and *M* ranging between 2 and 128

For a grid of points $\{(t_k,z_j)\}$ with $t_k=k\Delta t$, $k=1,\ldots ,K$, $\Delta t>0$ and $j=1,\ldots ,N$, we define the discrete $L^2(D_T)$ error for the semi-discrete scheme (16) as

$$\begin{aligned} E^1(\Delta t,h)=\left( \sum \limits _{k=1}^K\sum \limits _{j=1}^N(n(t_k,z_j)-\nu _j(t_k))^2h^3\Delta t_k\right) ^{1/2}, \end{aligned}$$

where $\nu (t)$ is the solution of the scheme for the functions introduced above. We set $\Delta t=0.01$ and in Fig. 1 we show the dependence in log-log scale of $E_h=E(0.01,h)$ with respect to the inverse of the cell size $M=1/h$.

**Fig. 2:**
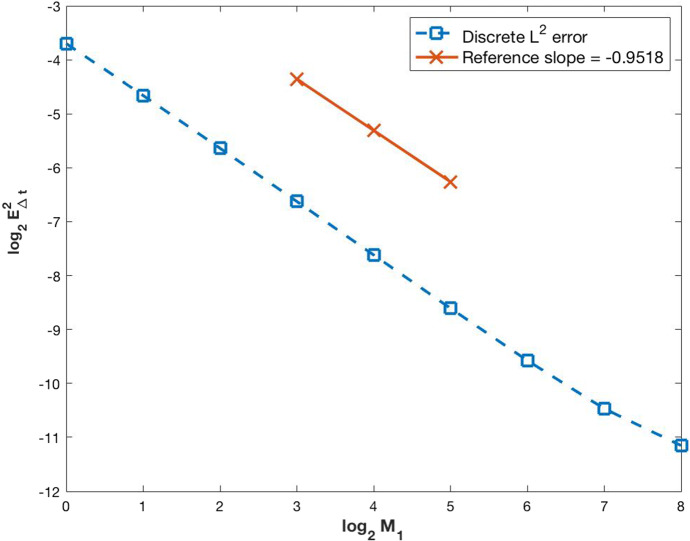
The discrete $L^2(D_T)$ error for the discrete scheme, for $T=10$ and $M_1$ ranging between 2 and 256

In the same way, we define the discrete $L^2(D_T)$ error for the discrete scheme (40) as

$$\begin{aligned} E^2(\Delta t,h)=\left( \sum \limits _{k=1}^K\sum \limits _{j=1}^N(n(t_k,z_j)-\nu ^k_j)^2h^3\Delta t_k\right) ^{1/2}, \end{aligned}$$

where $\nu ^k_j$ is the solution of (40). We set $h=1/50$ and plot the dependence of $E^2_{\Delta t}:=E^2(\Delta t,0.02)$ with respect to $\Delta t=1/M_1$, again in log-log scale (Fig. 2).

### Phenotypic dimorphism

We present now several examples illustrating the effect of the environment on a population and how the plasticity trait plays a role in surviving effects.

#### Monomorphic population

On the first place, we show the evolution of a population which is under the effects of natural selection and non-genetic epimuations, but without considering plasticity as a trait nor accounting for the environmental pressure. Specifically, we solve the problem (1–2) over the domain

$$\begin{aligned} \Omega =\{(x,y)\in [0,1]^2:(x-1)^2+(y-1)^2>1\}. \end{aligned}$$

This choice of domain represents the existence of a trade-off between traits and it is evidenced here by noticing that the individuals of the population which are close to the maximal value of one of the traits ($x=1$ or $y=1$), must forcibly be close as well to the minimal value of the other trait ($y=0$ or $x=0$). We take the growth rate as $r(x,y)=e^{-(x-0.1)^2-(y-0.1)^2}$, the death rate as $d(x,y)=0.5$, the diffusion parameters $a_{11}(\theta )=a_{22}(\theta )=10^{-6}$, and the drift terms $V(t,x,y)=(0,0)$. We take an initial condition (3) given by the expression

$$\begin{aligned} n_0(x,y)=a\mathbb {1}_{\{f(x,y)<1\}}e^{-\frac{1}{1-f(x,y)}}, \end{aligned}$$

with $f(x,y)=\frac{(x-0.25)^2+(y-0.25)^2}{(0.025)^2}$. We choose the value of *a* in such a way that $\rho _0=\int _{\Omega }n_0(x,y)dxdy=1$.

With this choice of $n_0$ we intend to represent a “strongly” monomorphic population. This is, a population where most of the individuals are concentrated around a single set of traits.

**Fig. 3:**
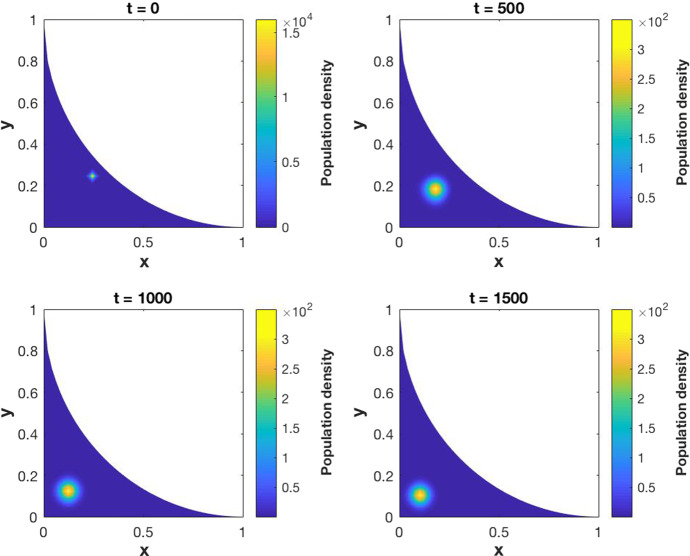
Evolution of a population only subjected to natural selection and non-genetic epimutations

We observe in Fig. 3 that the dominant phenotype slowly converges to the point which maximises the fitness, in this case, the point (0.1, 0.1), which maximises the growth rate *r*(*x*, *y*).

#### Dimorphism due to the effect of the environment

For a second example we keep the same parameters, but add a drift term accounting for the effect of the environment (biologically, a “cellular stress”). Specifically we choose

$$\begin{aligned} V(t,x,y)=10^{-3}\left( \mathbb {1}_{(y>x)}\begin{pmatrix} -1\\ 1 \end{pmatrix}+\mathbb {1}_{(y<x)}\begin{pmatrix} 1\\ -1 \end{pmatrix}\right) . \end{aligned}$$

Notice that we still are not including plasticity as a trait in our analysis. Also notice that the function *V*(*t*, *x*, *y*) is not continuous, while the results presented in previous sections needed *V*(*t*, *z*) to be smooth in order to ensure the existence and uniqueness of solution for the problem, as well as the convergence of the finite volume method. This is not a big issue, because the conditions of our problem allow us to use a density argument in order to extend our results to any $V(t,z)\in {\mathcal {C}}([0,T),L^2(D))$, and in any case all numerical approximations are smoothing approximations of the drift *V*.

The choice of *V* can be seen (and experimentally replicated) as a certain type of “training”: all the individuals of the population are “pushed” in the direction where they show their largest potential.

**Fig. 4:**
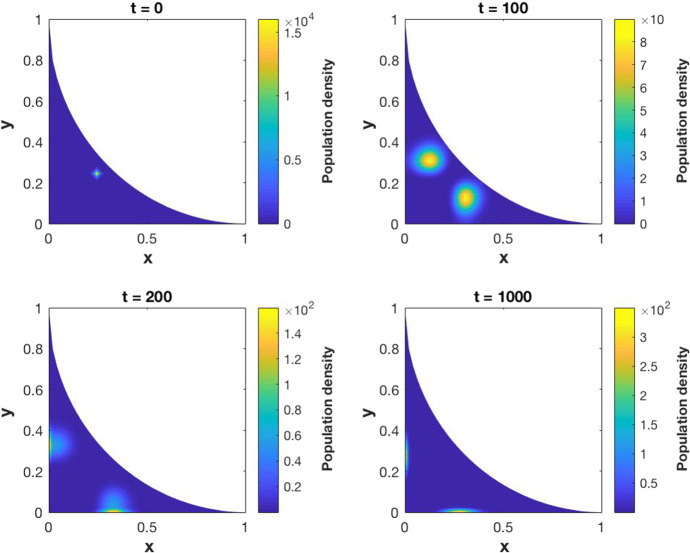
Evolution of a population under the effect of the environment

We can appreciate in Fig. 4 that adding a drift term resulted in the appearance of dimorphism: the final population is concentrated around two different trait configurations. This evolution into dimorphism due the action of environment, contrasts with the results obtained in Lorenzi and Pouchol (2020), where it was proved that dimorphism can occur in the absence of a drift term, given that the growth rate *r*(*x*, *y*) has several maximum point satisfying certain conditions.

#### Plasticity, environmental effect and dimorphism

We will now consider plasticity as a trait and modify the parameters from the previous examples accordingly. We first consider the growth rate as

$$\begin{aligned} r(x,y,\theta )=e^{-(x-0.1)^2-(y-0.1)^2}+e^{-(z-0.8)^2}, \end{aligned}$$

and keep the constant death rate $d(x,y,\theta )=0.5$. We consider the diffusion matrix

$$\begin{aligned} A(\theta )=\begin{pmatrix} (\theta +1)10^{-6}&{}0&{}0\\ 0&{}(\theta +1)10^{-6}&{}0\\ 0&{}0&{}10^{-6} \end{pmatrix}, \end{aligned}$$

and the drift term

$$\begin{aligned} V(t,z)=10^{-3}\theta \left( \mathbb {1}_{(y>x)}\begin{pmatrix} -1\\ 1\\ -x^2-y^2 \end{pmatrix}+\mathbb {1}_{(y<x)}\begin{pmatrix} 1\\ -1\\ -x^2-y^2 \end{pmatrix}\right) . \end{aligned}$$

This choice of *V* is similar to the one shown before, only that now differentiation imposes a cost on adaptability: the more specialised you are, the harder it gets to adapt to new situations. Notice that a higher plasticity increases the effect of non-genetic epimutations (given by the diffusion term) and stress induced mutations (given by the drift term). For the initial data (3) we take a function of the form

$$\begin{aligned} n_0(z)=a\left( \mathbb {1}_{\{f_1(z)<1\}}e^{-\frac{1}{1-f_1(z)}}+\mathbb {1}_{\{f_2(z)<1\}}e^{-\frac{1}{1-f_2(z)}}\right) , \end{aligned}$$

with $f_i(z)=\frac{\Vert z-z_i\Vert ^2}{(0.025)^2}$, $i=1,2$, $z_1=(0.25,0.25,0.25)$ and $z_1=(0.25,0.25,0.75)$. The value of *a* is again chosen in a way that the total population size over $D=\Omega \times [0,1]$ is equal 1.

**Fig. 5:**
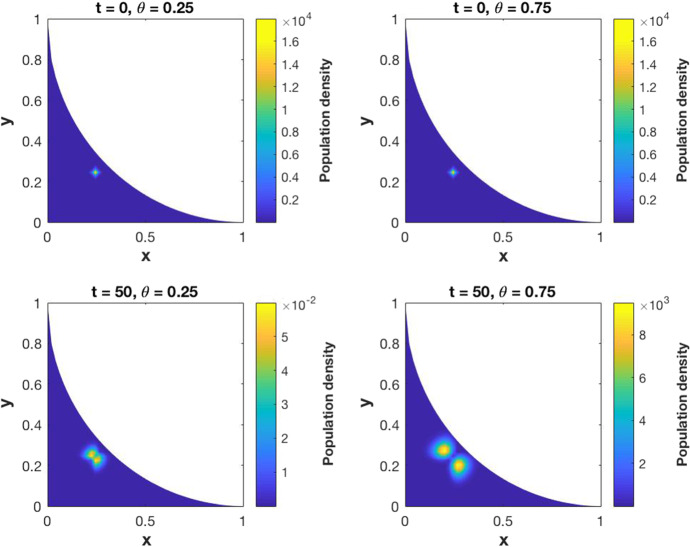
Evolution of two sub-populations with different levels of plasticity: initial stages

We observe in Fig. 5 that for this set of parameters, the sub-population with the lowest plasticity quickly gets extinct (notice the scale of the density values), while the emergence of dimorphism can be appreciated for the one with higher plasticity.

**Fig. 6:**
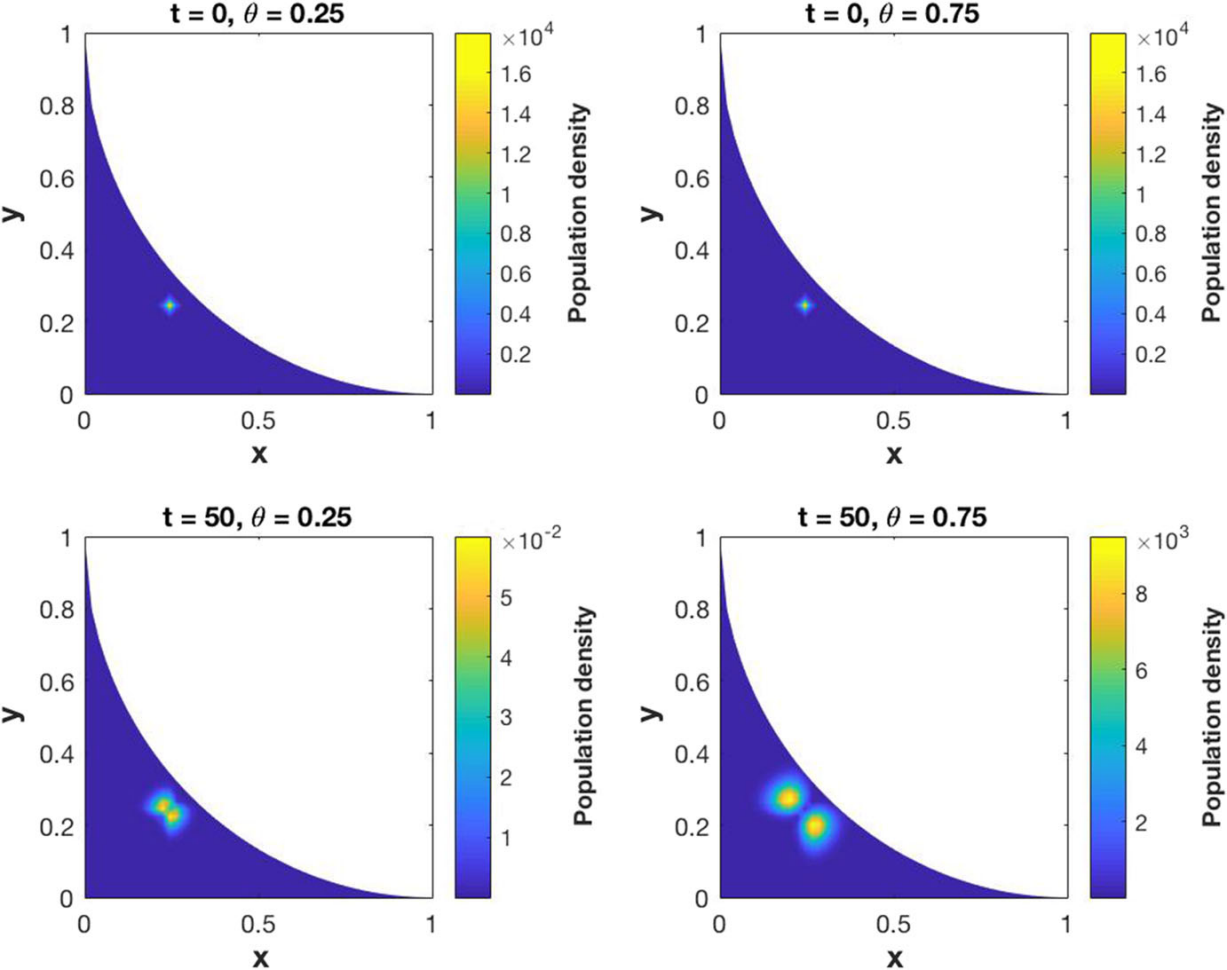
Evolution of two sub-populations with different levels of plasticity: final stages

This kind of behaviour persists up to the final stages of the evolution, when we can observe in Fig. 6 that the low plasticity sub-population is completely extinct while the high plasticity one has completed the differentiation process.

#### Another example of emergence of dimorphism

We present now a different example of emergence of dimorphism, but this time, not as a result of a response to the effect of the environment, but as a consequence of the existence of two maximum points for the growth rate. We will observe how a population initially concentrated around a single phenotype configuration, will evolve with time into a dimorphic population, in which each upcoming sub-population is more specialised and less plastic that in the initial configuration. For this purpose, over the domain $D=\Omega \times [0,1]$ we consider an initial density given by the expression

$$\begin{aligned} n_0(z)=a\mathbb {1}_{\{f(z)<1\}}e^{-\frac{1}{1-f(z)}}, \end{aligned}$$

with $f(z)=\frac{\Vert z-z_0\Vert ^2}{(0.025)^2}$, where $z_0=(0.25,0.25,0.5)$ and $\Vert \cdot \Vert$ is the euclidean norm. We choose the value of *a* in such a way that $\rho _0=\int _{D}n_0(z)=1$.

We set the growth rate and the death rate as

$$\begin{aligned} r(x,y,\theta )&=\mathbb {1}_{\{y>x\}}e^{-(0.1-x)^2-(0.9-y)^2}+\mathbb {1}_{\{x\geqslant y\}}e^{-(0.1-y)^2-(0.9-x)^2},\\ d(x,y,\theta )&=\frac{1}{2}. \end{aligned}$$

We choose the diffusion matrix

$$\begin{aligned} A(\theta )=\begin{pmatrix} (\theta +1)10^{-6}&{}0&{}0\\ 0&{}(\theta +1)10^{-6}&{}0\\ 0&{}0&{}10^{-6} \end{pmatrix}, \end{aligned}$$

and finally the drift term

$$\begin{aligned} V(t,z)=10^{-3}\theta \begin{pmatrix} -y\\ -x\\ -(x+y) \end{pmatrix}. \end{aligned}$$

This time, the “push” towards specialisation imposed by *V* is inversely proportional to the current set of traits (individuals with traits (*x*, *y*) are specialising with a rate proportional to $(-y,-x)$. The growth rate was chosen in such a way that it satisfies the sufficient conditions given in Lorenzi and Pouchol (2020) in order to guarantee the appearance of phenotypic polymorphism.

**Fig. 7:**
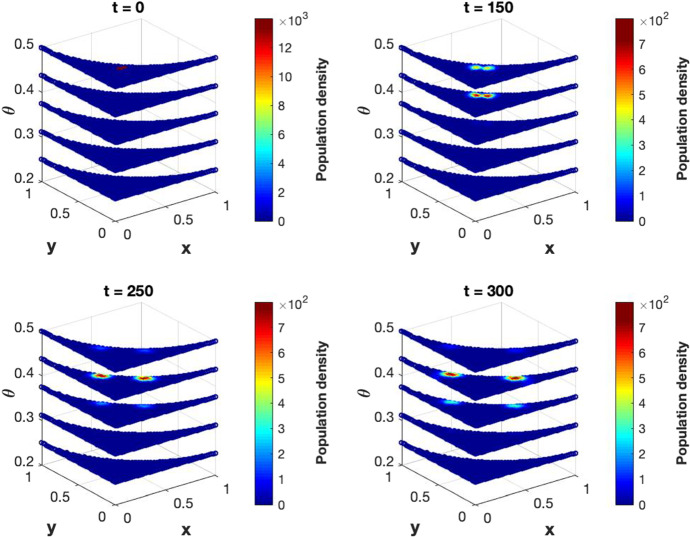
Initial stages of the population density for different values of $\theta$: The differentiation process starts. At around $t=250$ (bottom left) most of the population has already concentrated around the plasticity level $\theta =0.4375$ and around $t=300$ (bottom right) we observe that the migration towards a less plastic state continues

We show in Fig. 7 that initially the population is concentrated around the phenotype $z_0=(0.25,0.25,0.5)$, and gradually starts differentiating while loosing plasticity.

**Fig. 8:**
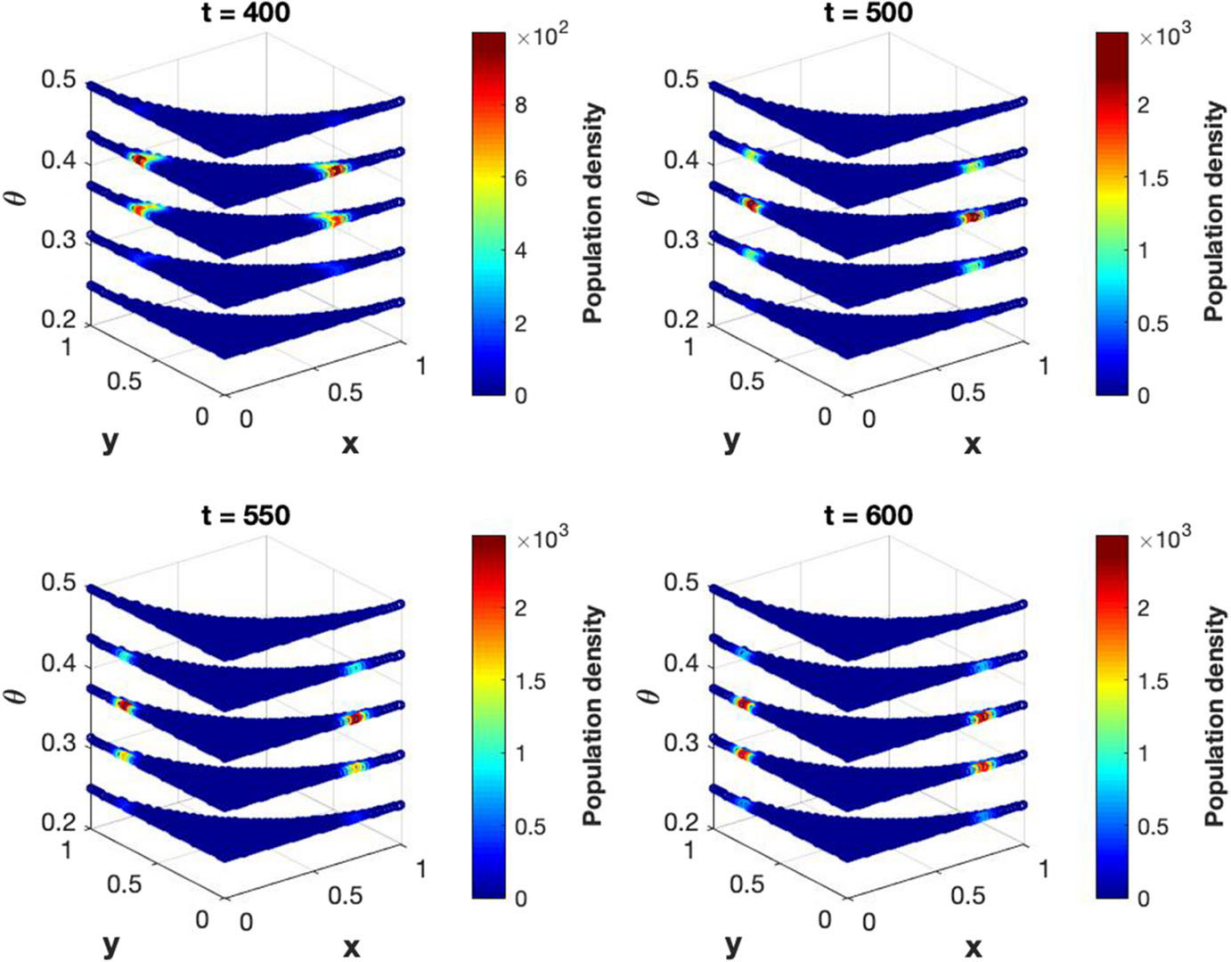
Intermediate stages of the population density for different values of $\theta$: While the differentiation process continues, we observe further loss in plasticity. Around $t=500$ (top right) most of the population has reached $\theta =0.375$ and at subsequent times the migration continues

As the two new sub-populations become more and more differentiated, the loss in plasticity becomes more evident, and we see in Fig. 8 that most of the mass is migrating towards less plastic states, while the differentiation process continues.

**Fig. 9:**
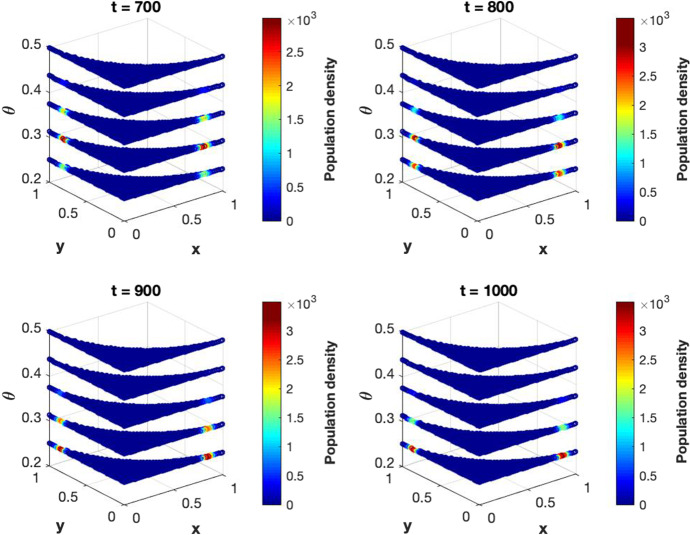
Final stages of the population density for different values of $\theta$: Around $t=900$ (bottom left) the differentiation process is over and most of the population has reached the plasticity level $\theta =0.25$. At $t=1000$ (bottom right) we observe that the population concentrated around any other level of plasticity is almost extinct, and only the one around $\theta =0.25$ survives

Finally we observe in Fig. 9 that once the sub-populations are fully specialised, the concentration process continues and at the final stage $t=1000$ we have a dimorphic population which is more specialised but less plastic that the initial one.

Overall, in the absence of a drift term, the effects of the growth and death rate, with small (or zero) diffusion has been widely studied, for example, see (Perthame 2006; Pouchol and Trélat 2018; Lorenzi and Pouchol 2020). In general, the points where the population concentrates are entirely determined by the reaction part, while the diffusion coefficients determine how concentrated the population is.

The shape of the domain $\Omega$ directly affects where the fitness function (which depends on the birth and death rates) attains its maxima, therefore, affecting as well where the concentration phenomena occurs.

The choice of initial state does not appear to affect the final configuration, but rather the dynamics of the population during the evolution in time. We have shown that if we start with an already concentrated population, we witness the continuous “migration” of the the population towards the fittest state.

Finally, if we consider the presence of the advection term, we observe that it may affect not only the amount of selected sets of traits, but their positions and the dynamics of convergence towards them as well.

## Concluding remarks

The validity of the model we constructed is strengthened by the different evolutionary mechanisms described in Wagner et al. (2019), where the authors focus their attention on Stress-Induced Evolutionary Innovation, and compare it to plasticity-based models, in particular the Plasticity First Hypothesis. Quoting the authors: “SIEI and PFH are not competing models but explain different kinds of evolutionary processes that are sometimes distinct and sometimes combined over evolutionary time”. Similar mechanisms were taken into consideration in the construction of our model, environmental stress (aka environmental pressure, biologically, at the single-cell level, “cellular stress”) in the form of an advection term and and mutations thanks to plasticity in the form of a diffusion term, both accompanied by natural selection in the form of reaction term.

It is important to highlight the novelty that represents the inclusion of plasticity as a trait, which has not been considered before (with the exception of Bouin et al. 2012; Bouin and Calvez 2014 and the subsequent works on the cane toad spreading rate, where a similar structural variable is used, but to denote spatial diffusivity, and not adaptability potential, as in our case). Another novelty is the inclusion of constraints between traits modelled through a certain relation between the structural variables. Most of the previous work, and in particular (Chisholm et al. 2015), have considered the variable space as the entire unit square, or all of ${\mathbb {R}}^d$, $d\in {\mathbb {N}}$, disregarding the existence of constraints between different traits, and the possible effects on the dynamics of the population this might have.

The finite volume method offers a powerful tool in order to numerically approximate the solution of integro-differential or reaction-diffusion equations, such as the one treated in the present paper. The preservation of the structure of the original problem at a semi-discrete level and the excellent approximation for the non-local terms are just two of the reasons why we chose this method. This way, we were able to obtain two numerical schemes in order to approximate the solution for an evolution problem modelling bet hedging strategies.

We proved the existence and uniqueness of solutions for such schemes, and constructed sequences of functions converging to the solution of the original problem. We approximated the convergence error by establishing a comparison with an exact solution. It is worth mentioning that the constructive character of the proofs may provide new and interesting tools in order to obtain further theoretical results.

After simulating various situations, we observed different ways in which a population can respond to external stress, depending on the plasticity levels of its individuals. A highly plastic sub-population can quickly adapt to its surrounding environment, guaranteeing this way its survival, while a less plastic sub-population might go extinct under the same external factor. Another strategy consists in “trading” some of the plasticity by a higher differentiation level.

Furthermore, the emergence of dimorphism as a consequence of external stress, not only is an interesting alternative to the previously established results from Lorenzi and Pouchol (2020), but also shows that bet-hedging strategies are a suitable response to (abrupt) external changes in the environment, and, at the same time, a possible way to survive them. It is fair mentioning that, throughout all the simulations, the symmetry hypothesis required in the reference (Lorenzi and Pouchol 2020) in order to observe dimorphism were respected. It remains to establish what are the essential conditions that will lead to the appearance of dimorphism when an advection term is present.

We thus provided here a rigorous model for the study of the emergence of dimorphism, an event that is likely to have been at the evolutionary origin of multicellularity by divergence of phenotypes and may thus provide a rationale for a renewed conception of animal evolution towards multicellular organisms, and, more pragmatically and consistently with the atavistic theory of cancer, for a possible origin of phenotype bet hedging in cancer cell populations.

Bet hedging in cancer cell populations is indeed a strategy susceptible to yield maximal probabilities of survival to a plastic cell population exposed to life-threatening insults such as by drugs or other deadly therapies. The modelling setting presented here may thus help in the future to test and optimise combined anticancer therapies involving chemotherapies, targeted therapies, and - what is likely still ahead of us for the present time - possible control of cell plasticity by epigenetic drugs.

## Data Availability

Not applicable.
